# Dynamic Perfusion and Cell Seeding Density Govern Remodeling and Mechanical Maturation of Bioprinted Collagen Constructs

**DOI:** 10.3390/gels12080698

**Published:** 2026-08-05

**Authors:** Denisa Kaňoková, Jana Matějková, Martin Otáhal, Jan Žigmond, Nina Skalová, Margit Žaloudková, Monika Šupová, Roman Matějka

**Affiliations:** 1 Department of Biomedical Technology, Faculty of Biomedical Engineering, Czech Technical University in Prague, Sitna 3105, 272 01 Kladno, Czech Republic; kanokden@cvut.cz (D.K.); jana.matejkova@cvut.cz (J.M.); jan.zigmond@fbmi.cvut.cz (J.Ž.); 2 Department of Natural Sciences, Faculty of Biomedical Engineering, Czech Technical University in Prague, Sitna 3105, 272 01 Kladno, Czech Republic; martin.otahal@fbmi.cvut.cz; 3 Department of Health Care and Population Protection, Faculty of Biomedical Engineering, Czech Technical University in Prague, Sitna 3105, 272 01 Kladno, Czech Republic; skalonin@cvut.cz; 4 Department of Composites and Carbon Materials, Institute of Rock Structure and Mechanics, Czech Academy of Sciences, 182 09 Prague, Czech Republic; zaloudkova@irsm.cas.cz (M.Ž.); supova@irsm.cas.cz (M.Š.)

**Keywords:** bioprinting, collagen hydrogels, dynamic perfusion, cell seeding density, remodeling, mechanical properties, extracellular matrix organization

## Abstract

Hydrogel-based three-dimensional culture systems are widely used in tissue engineering; however, their maturation is often limited under static conditions. This study investigates the combined effects of dynamic perfusion and initial cell seeding density on remodeling behavior and mechanical properties of bioprinted collagen hydrogel constructs, with additional assessment of smooth muscle cell-like (SMC) differentiation. Rectangular constructs (30 × 15 × 1.5 mm) were fabricated using high-concentration collagen (30 mg/mL) with cell densities of 10 and 20 million cells/mL. MCDB- and DMEM-based media were first compared using growth curves, leading to the selection of MCDB differentiation medium for subsequent experiments. Constructs were then cultured statically or under dynamic perfusion (20 mL/min) for up to 7 days. Remodeling was evaluated by monitoring changes in construct dimensions over time. Static constructs exhibited non-uniform deformation and rolling, whereas dynamically perfused samples retained their geometry and underwent homogeneous contraction. Mechanical testing revealed a transition from stiff and brittle to more compliant and ductile behavior, with preserved load-bearing capacity at large strains. Remodeling and mechanical outcomes were strongly influenced by cell density and culture medium, with differentiation conditions promoting more stable constructs. These changes were accompanied by increased expression of smooth muscle–related markers under dynamic culture. Overall, dynamic perfusion and cell seeding density jointly govern remodeling and mechanical maturation of bioprinted collagen constructs, highlighting their importance for functional hydrogel-based tissue development.

## 1. Introduction

Type I collagen is one of the key hydrogels in tissue engineering due to its role as the most abundant extracellular matrix (ECM) protein in native tissues and its excellent biocompatibility and intrinsic cell-adhesive properties [1,2]. In the form of hydrogels, collagen closely mimics the hydrated and viscoelastic nature of soft tissues, providing a three-dimensional (3D) microenvironment that supports cell attachment, migration, and cell–matrix signaling [3,4]. Owing to these properties, collagen hydrogels are widely used as model systems for studying tissue formation and remodeling as well as for the fabrication of engineered tissue constructs.

Compared to conventional two-dimensional (2D) monolayer cultures, 3D culture models better recapitulate the complex spatial organization of cells and the dynamic interactions between cells and the surrounding ECM that are essential for tissue-specific function [3,4]. However, these advantages are accompanied by several challenges, including increased technical complexity, higher costs, and reduced reproducibility [3]. One of the most critical limitations of 3D culture systems is restricted mass transport. In avascular constructs, the diffusion of oxygen and nutrients is typically limited to a thickness of approximately 200 µm to 2 mm, which can lead to the formation of hypoxic or necrotic regions within the construct core [5,6].

To improve the mechanical integrity of collagen-based constructs and to mitigate the rapid cell-induced contraction characteristic of low-concentration hydrogels, high-concentration collagen bioinks (>10 mg/mL) have been increasingly employed [6,7]. In such dense matrices, embedded cells actively participate in matrix remodeling through degradation of the initial collagen network and its subsequent replacement with newly synthesized extracellular matrix components [5]. This cell-mediated remodeling is particularly relevant in cardiovascular tissue engineering, where extensive extracellular matrix reorganization is required to achieve mechanically robust, load-bearing tissue constructs. Dynamic culture approaches, including active media perfusion, have been introduced to further support the maturation of high-concentration collagen constructs by alleviating mass transport limitations inherent to static culture [7]. By improving nutrient and oxygen availability throughout the construct volume, perfusion culture has been associated with improved cell viability and more homogeneous remodeling of the collagen matrix [7]. A detailed understanding of how these dynamic conditions influence cell-driven remodeling processes is therefore essential for the rational design of mechanically functional collagen-based tissue constructs.

In addition to culture conditions, intrinsic design parameters such as initial cell density further modulate the remodeling behavior and functional maturation of collagen-based constructs. Initial cell seeding density has been identified as a key determinant of both matrix remodeling kinetics and the development of mechanical properties in cell-laden collagen constructs [8]. Higher initial cell densities are associated with markedly enhanced hydrogel contraction; for example, high-density fibroblast-laden constructs have been reported to shrink to as little as 0.08% of their initial surface area, compared to approximately 3% in constructs seeded at lower densities [9]. In parallel, increased cellular density promotes mechanical reinforcement of the construct through intensified extracellular matrix (ECM) synthesis and accumulation, resulting in progressive increases in stiffness and strength over time [8,10]. Cell density also modulates biological outcomes, with higher local densities generally favoring proliferation, whereas lower densities tend to promote differentiation [9]. However, excessive cell densities may adversely affect viability as a consequence of limited oxygen and nutrient transport within dense hydrogel matrices, underscoring the need for balanced construct design [8].

Vascular smooth muscle cells (SMCs) play a central role in the remodeling of three-dimensional collagen hydrogels by actively reorganizing and degrading extracellular matrix (ECM) structures to recapitulate features of native vascular tissue [11]. This process is driven by cell-generated forces that enable displacement of collagen fibers over distances exceeding hundreds of micrometers [12]. While remodeling induced by isolated cells is often relatively isotropic, collective SMC behavior gives rise to pronounced anisotropic matrix compaction [12]. These structural changes are largely mediated by matrix metalloproteinase (MMP) activity, which facilitates degradation of the original collagen network and its replacement with newly synthesized ECM components [11]. In high-concentration collagen bioinks, such cell-mediated remodeling is associated with construct shrinkage and progressive mechanical reinforcement, supporting the maturation of the hydrogel into a mechanically robust tissue-like structure [7].

Despite increasing interest in dynamic culture strategies for hydrogel-based tissue engineering, the interplay between perfusion-driven remodeling, mechanical behavior, and smooth muscle–oriented differentiation in bioprinted collagen constructs remains insufficiently understood. In particular, systematic comparisons of static and dynamic culture under well-defined material composition, construct geometry, and initial cell seeding density are still limited.

Therefore, this study aimed to investigate how dynamic perfusion influences remodeling behavior, mechanical maturation, and smooth muscle–like differentiation of stromal cells in bioprinted porcine collagen hydrogels. Static and dynamic culture conditions were compared over a 7-day cultivation period using controlled construct geometry and collagen composition. Additionally, the effects of initial cell seeding density and culture medium on dimensional remodeling, mechanical properties, and cytoskeletal organization were systematically evaluated to identify key factors that govern construct development.

## 2. Results and Discussion

### 2.1. Selection of Culture Medium—Growth Curve Analysis, Metabolic Activity, and Production of Differentiation Markers

In the context of smooth muscle cell-like (SMC) differentiation, DMEM-based media, typically supplemented with 10% FBS, are characterized as a medium that maintains a differentiated phenotype or preserves the cells’ contractile phenotype [13], while also being recommended for initial expansion of human mesenchymal stem cells (hMSCs) with minimal effect on differentiation induction, making it suitable for initial cell expansion without inadvertently directing the cells toward the SMC lineage [14]. In contrast, MCDB 131, originally developed for endothelial cell culture, is nutritionally richer and has a lower serum content, which allows for better control of culture conditions [15]. It is specifically used for the culture of human aortic smooth muscle cells (HA-VSMC) [16]. SMCs cultured in MCDB exhibit significantly higher rates of proliferation and migration than in DMEM-based medium [13]; however, their use results in a decrease in the expression of differentiation markers (de-differentiation) in SMCs [13]. Based on these characteristics, four culture media formulations were evaluated to identify the most suitable conditions for subsequent experiments.

**Figure 1:**
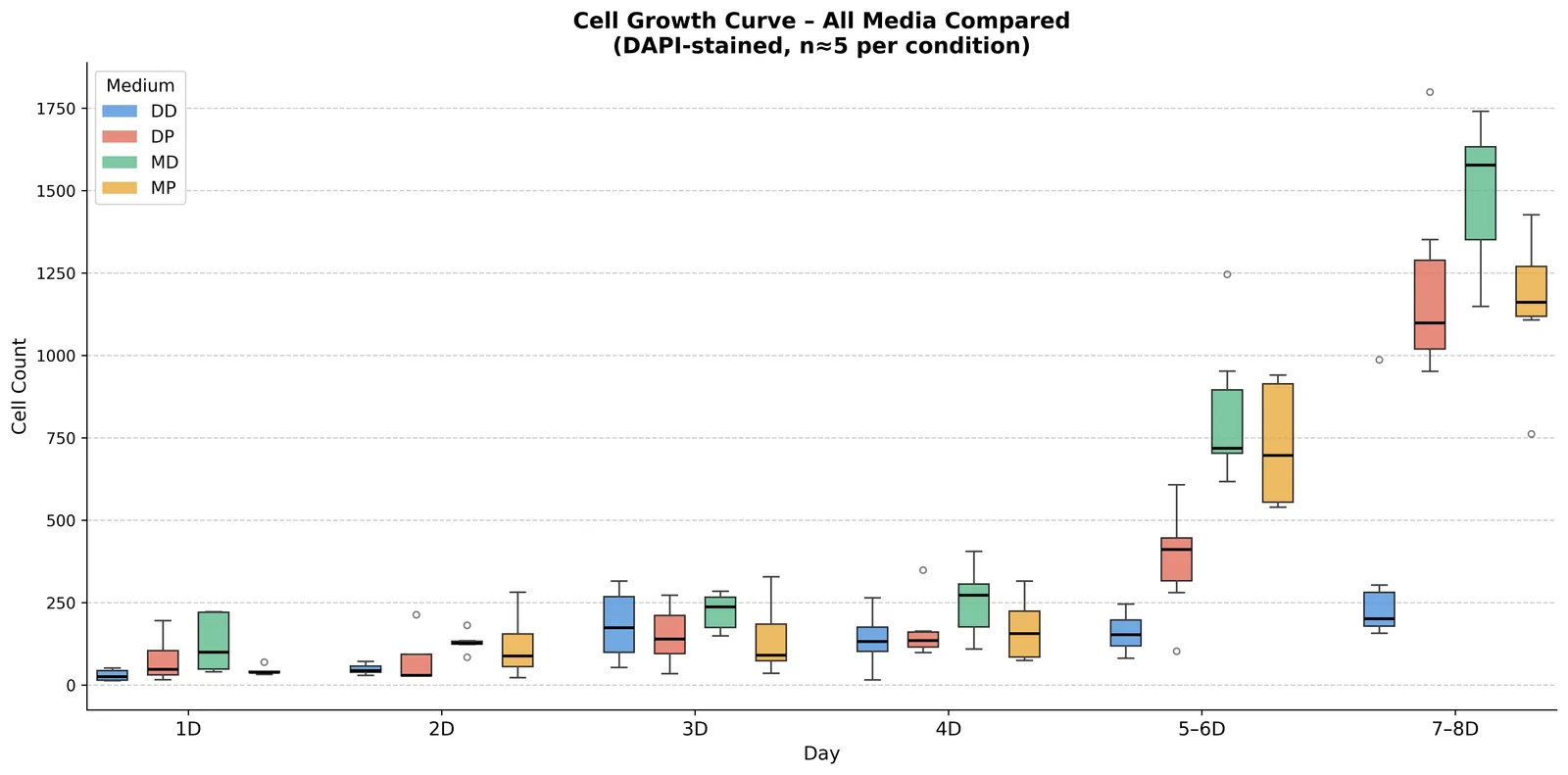
Growth curves, representing cell counts measured over an 8-day cultivation period, were used to assess and compare the performance of different culture media (DP—proliferative DMEM/F12, DD—differentiation DMEM/F12, MP—proliferative MCDB, MD—differentiation MCDB) for subsequent experiments.

To assess cell proliferation over time, a growth curve assay was performed, monitoring cell numbers across all four media formulations from day 1 to day 7. Wharton’s jelly-derived stromal cells (WJCs) cultured in all four media (DP—proliferative DMEM, DD—differentiation DMEM, MP—proliferative MCDB, MD—differentiation MCDB) exhibited an increase in cell count from day 1 to day 7 (Figure 1). The highest cell numbers at day 7 were observed in MD and MP media, with median values of 1585 and 1321, respectively. Cells cultured in DP medium also showed a substantial increase from day 3 onward, reaching a median count of 1052 by day 7, approaching the levels observed in MD and MP conditions. The individual growth curves for each medium are shown in Supplementary Materials part A in Figures S1–S3.

In contrast, cells cultured in DD medium formed aggregates during cultivation and subsequently detached from the culture surface, resulting in consistently lower cell counts across all time points. The median cell count in DD cultures did not exceed 183 at any time point. A single measurement at day 7 (987 cells) was identified as a potential outlier.

**Figure 2:**
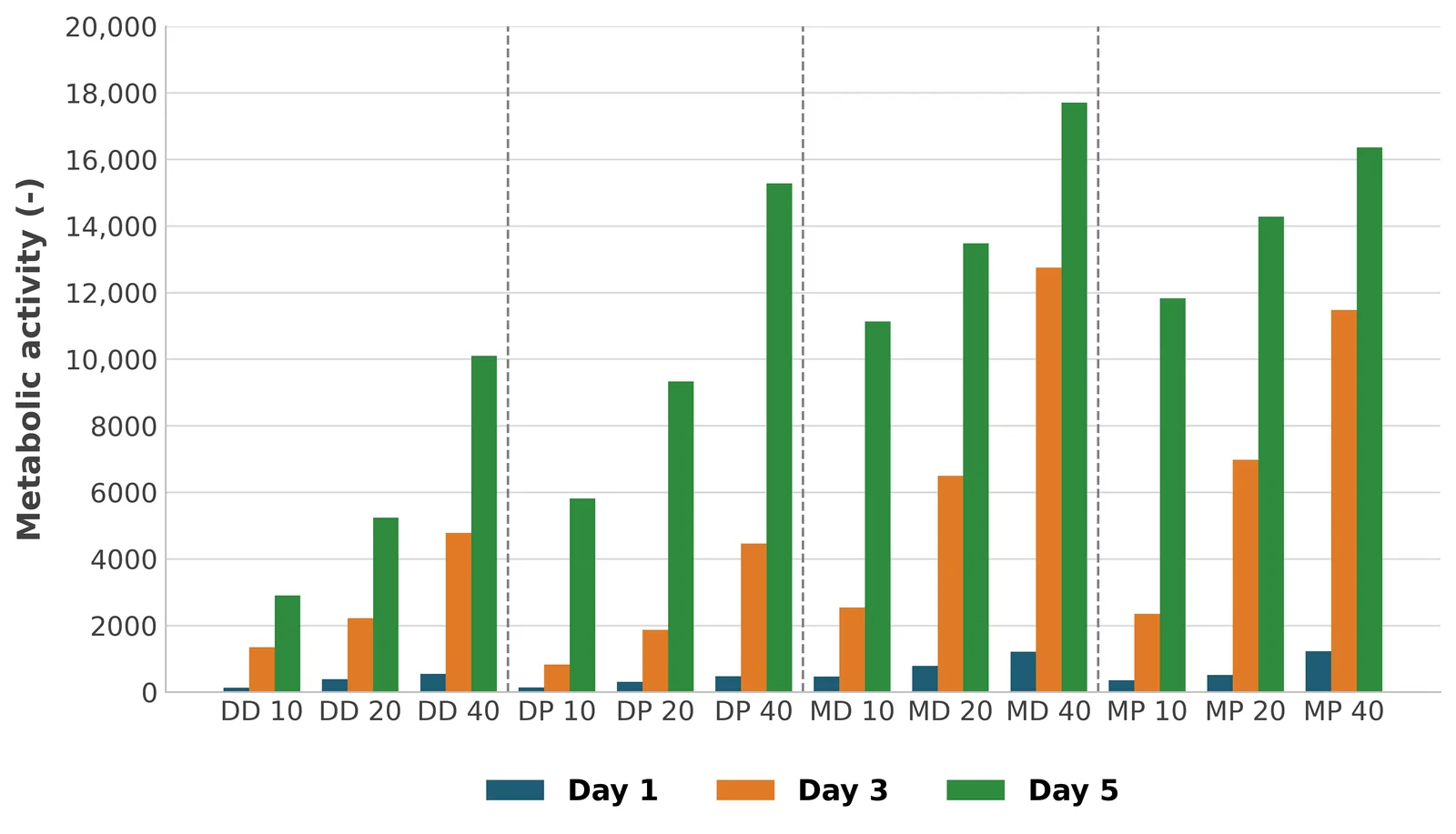
Metabolic activity of 2D cultures in different media (DP—proliferative DMEM/F12, DD—differentiation DMEM/F12, MP—proliferative MCDB, MD—differentiation MCDB) and seeding densities 10, 20, and 40 thousand/cm^2^ from 1 to 5 days of cultivation.

Metabolic activity measurements in 2D cultures (Figure 2) revealed an increase over time across all conditions, consistent with the observed growth curves. Cells cultured in MP and MD media exhibited the highest metabolic activity values, particularly at higher seeding densities, whereas significantly lower activity was detected in DD conditions throughout the entire cultivation period. The influence of initial cell density was evident across all media, with higher seeding densities resulting in increased metabolic activity at later time points.

**Figure 3:**
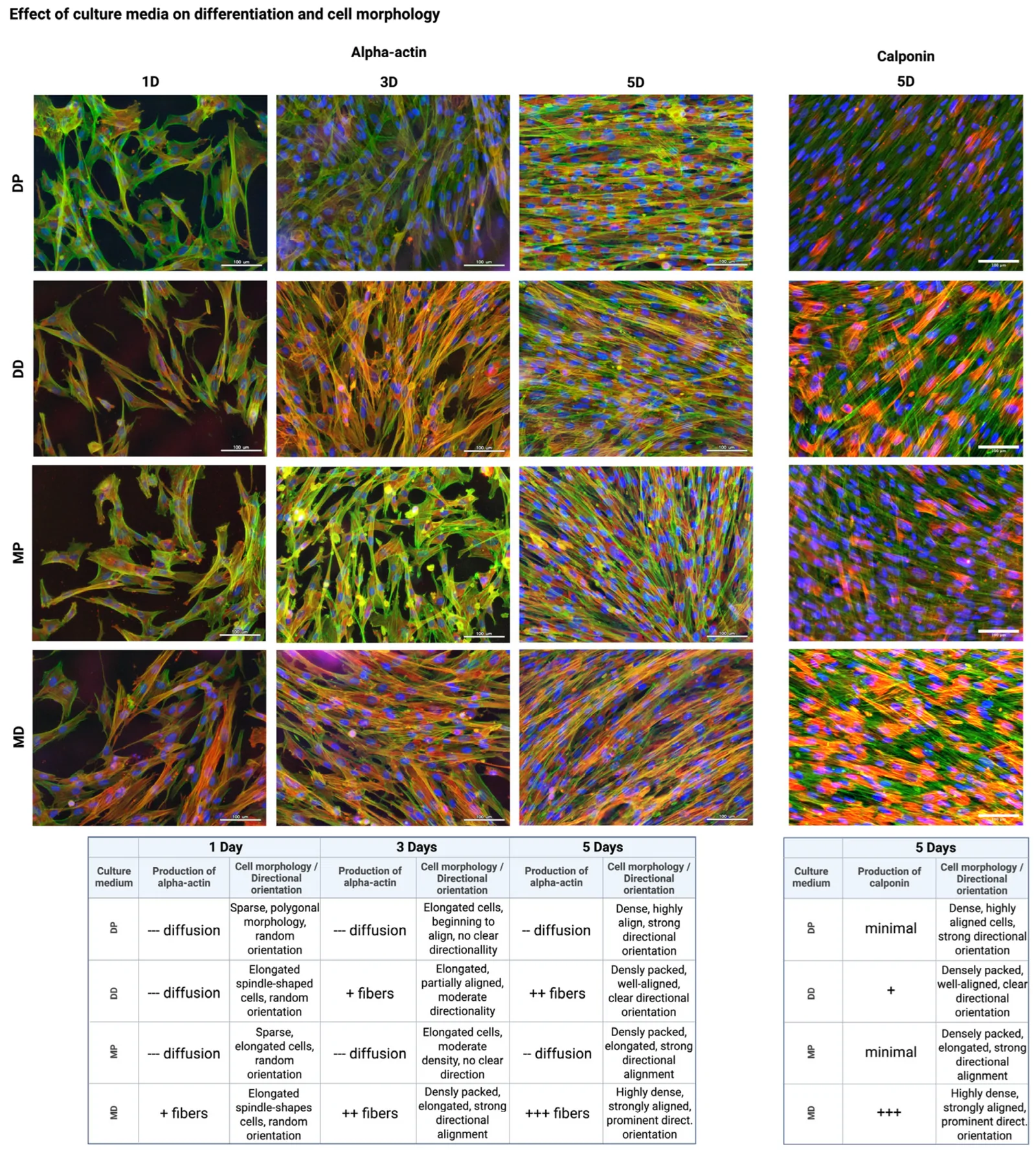
Immunofluorescence characterization of smooth muscle–related marker expression and cytoskeletal organization in WJ-MSCs cultured in proliferation (DP, MP) and differentiation (DD, MD) media over 1, 3, and 5 days. Cells were stained for α-smooth muscle actin (α-SMA, red) or calponin (red), F-actin (green), and nuclei (DAPI, blue). Images were acquired at 4× magnification using a ThorLabs CERNA microscope equipped with an Olympus Plan Fluoro objective. The summary table below reflects the temporal progression of α-SMA and calponin expression and cell orientation. Created in BioRender. Matějka, R. (2026) https://BioRender.com/a6rerme.

To assess the differentiation capacity of Wharton’s jelly-derived mesenchymal stem cells (WJ-MSCs) toward the smooth muscle cell (SMC) lineage, cells were cultured for up to 5 days in all four culture media formulations, consisting of DMEM- or MCDB-based culture media, which were formulated as either SMC differentiation medium or standard proliferation medium, and subsequently stained for an established SMC marker—alpha-smooth muscle actin (α-SMA) or calponin—alongside DAPI and F-actin (Figure 3).

Immunofluorescence imaging at 10× magnification across all four media formulations revealed progressive changes in both α-SMA and calponin expression, as well as cell morphology, with markedly enhanced α-SMA and calponin expression in cells maintained under differentiation conditions compared to those cultured in proliferation medium. On day 1, cells in all conditions displayed a sparse distribution with an elongated, spindle-shaped morphology and random orientation, except for MD, which already showed early α-SMA fiber formation. By day 3, cells cultured in differentiation media (DD and MD) exhibited increasingly organized actin stress fibers and partial directional alignment, with MD showing notably stronger and more structured α-SMA signal compared to DD. By day 5, MD-cultured cells displayed the most prominent α-SMA and calponin expression with highly dense, strongly aligned fiber organization, indicative of SMC-like differentiation. In the differentiation medium group, α-SMA and calponin staining displayed a prominent fluorescent signal with a well-organized, elongated fibrillar pattern, indicative of actin stress fiber assembly characteristic of contractile phenotype. DP and MP media, both proliferative formulations, showed consistently diffuse α-SMA signal across all time points with no clear fiber formation, while cells reached high density with strong directional alignment by day 5, reflecting active proliferation rather than differentiation. The initial seeding density had no substantial effect on the overall differentiation response or morphological organization, as cells across all tested densities followed comparable trends within each medium condition, suggesting that the culture medium composition was the dominant factor governing cell behavior under the tested conditions.

Collectively, growth curve analysis and metabolic activity measurements showed consistent trends across all tested media, confirming reliable assessment of cell behavior. Cell numbers and metabolic activity increased over time in all conditions, indicating continuous proliferation. These findings demonstrate that MCDB-based media supported superior proliferative activity compared to DMEM-based formulations, and that differentiation medium effectively promoted the phenotypic transition of WJ-MSCs toward a smooth muscle-like cell type, as evidenced by the upregulation and reorganization of α-SMA within 5 days of induction. MD and MP media supported the highest growth and metabolic activity, whereas DD medium consistently showed reduced values, accompanied by cell aggregation and detachment, which is consistent with its differentiation-promoting composition and the known tendency of SMCs in differentiation conditions to adopt a contractile, less proliferative phenotype. Higher initial cell densities led to increased metabolic activity at later time points, likely reflecting enhanced cell–cell interactions, although potential transport limitations at higher densities cannot be excluded. Based on these findings, the MCDB-based media were selected for subsequent experiments.

### 2.2. Evaluation of Flow Direction on Cell Morphology in Dynamic Conditions

**Figure 4:**
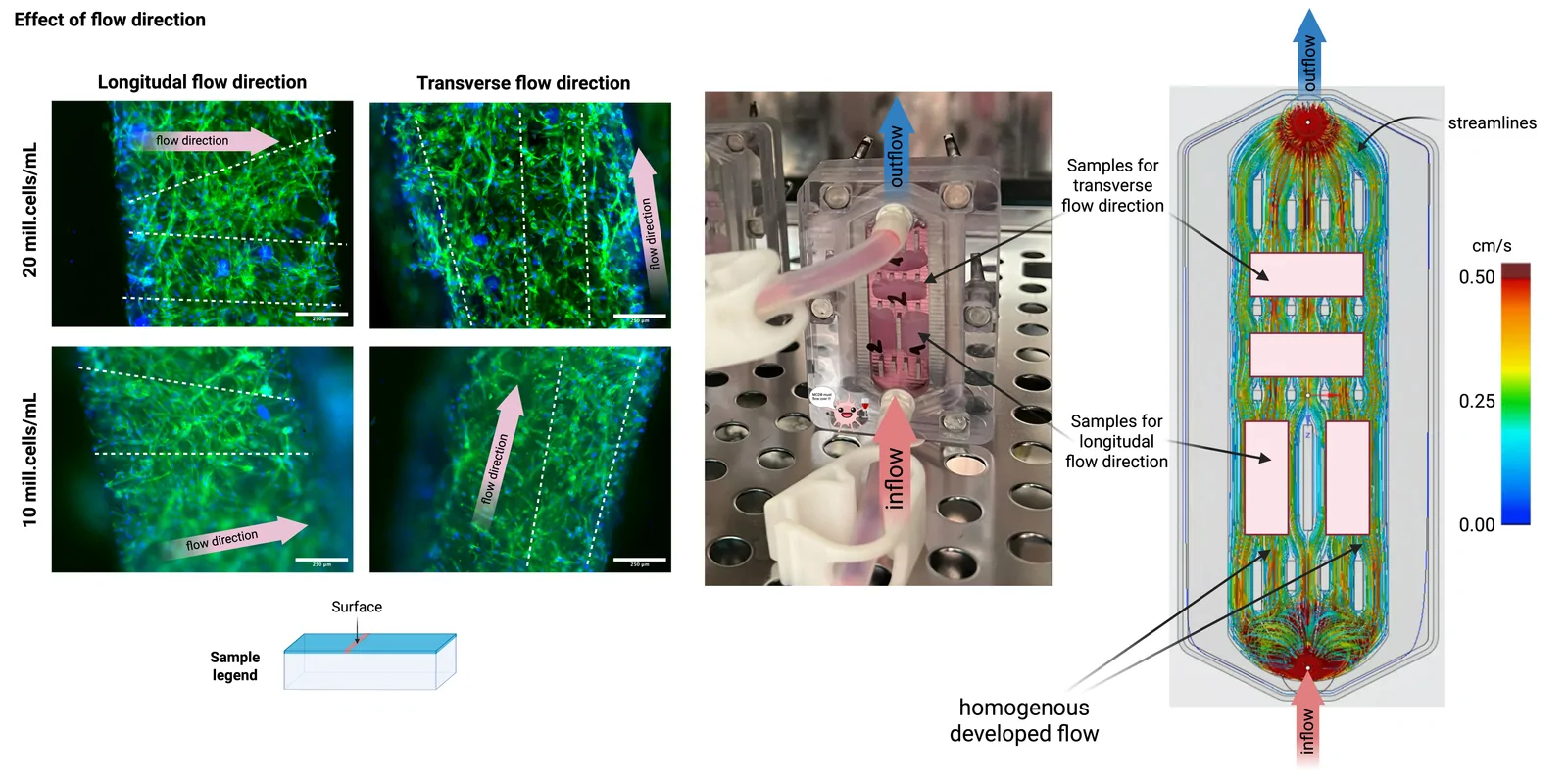
Effect of flow direction on cell alignment in bioprinted constructs under dynamic culture conditions. Fluorescence images (DAPI, blue; F-actin/phalloidin, green) of bioprinted collagen-based constructs at two seeding densities (10 and 20 million cells/mL) cultured for 4 days under longitudinal and transverse flow directions. Dashed white lines and pink arrows indicate the predominant direction of cell alignment and flow direction, respectively. (**Center**): photograph of the bioreactor chamber showing sample placement for transverse (upper position) and longitudinal (lower position) flow orientation relative to the inflow and outflow ports. (**Right**): computational fluid dynamics (CFD) simulation of flow streamlines and velocity distribution within the bioreactor chamber, confirming the development of homogeneous flow conditions across sample positions. Flow velocity scale bar: 0–5.79 cm/s. Created in BioRender. Matějka, R. (2026) https://BioRender.com/33imi67.

To investigate the effect of flow direction on cell alignment within bioprinted constructs, samples (15 × 8 × 1.5 mm) were placed in the bioreactor in two orientations—longitudinal and transverse relative to the flow direction—and cultured for 4 days in MD medium (Figure 4). Computational fluid dynamics (CFD) simulation confirmed the development of homogeneous flow conditions across the sample positions within the bioreactor chamber. Fluorescence imaging of F-actin revealed that flow direction had a pronounced effect on intracellular cytoskeletal organization, with cells aligning along the predominant flow axis in both conditions. In longitudinally oriented samples, cells displayed alignment parallel to the flow direction, whereas in transversely oriented samples, cells adopted a diagonal to perpendicular alignment relative to the flow. Both cell densities (10 and 20 million cells/mL) responded similarly to the applied flow, confirming that the observed alignment was driven by the mechanical stimulus of fluid flow rather than cell density.

To ensure reproducibility and consistency across all subsequent dynamic culture experiments, longitudinal flow direction was selected as the standard orientation. Furthermore, as the constructs undergo substantial remodeling and volumetric shrinkage during cultivation, pinpoints were used to secure the samples within the bioreactor, preventing rotation or displacement during the shrinkage process and ensuring maintained alignment throughout the cultivation period.

### 2.3. Cell Distribution in Samples Under Different Conditions

**Figure 5:**
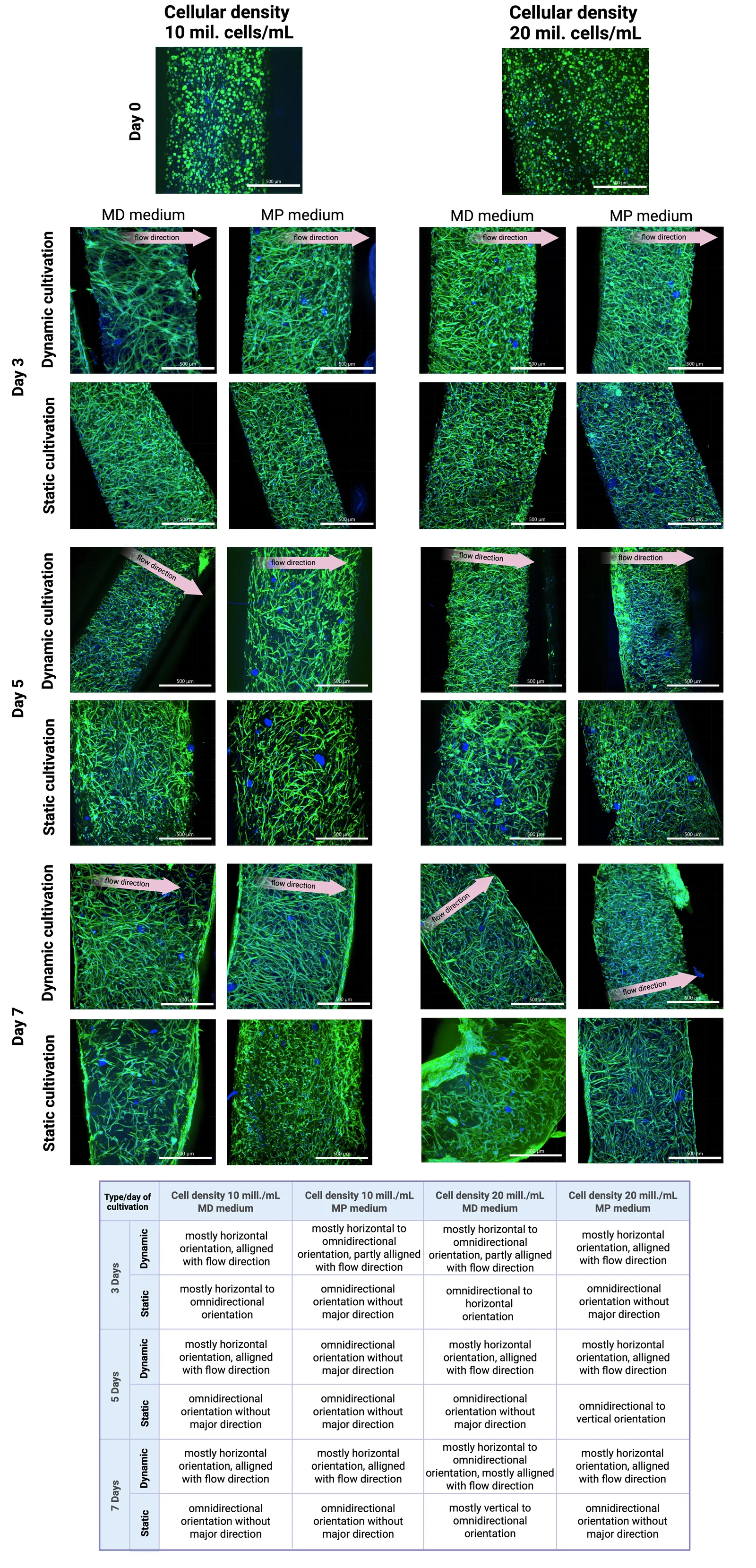
Fluorescence images of nuclei (blue) and actin cytoskeleton (green) in bioprinted collagen constructs cultured under static (stat) and dynamic perfusion (dyn) conditions in proliferative (MP) and differentiation (MD) MCDB media over time (days 0, 3, 5, and 7), including actin orientation analysis. Constructs with different initial cell densities (10 and 20 million cells/mL) are shown as internal sections. Semi-quantitative evaluation of actin organization is presented in the table below. Arrows indicate flow direction. Scale bars: 100 µm. Created in BioRender. Matějka, R. (2026) https://BioRender.com/c7oyunk.

As shown in Figure 5, cytoskeletal organization was strongly influenced by culture conditions, initial cell seeding density, and time of cultivation. On day 0, cells were distributed relatively uniformly with no evident alignment in either group. Over time, distinct differences emerged between static and dynamic culture conditions.

At lower cell density (10 million cells/mL), statically cultured constructs maintained a relatively sparse and disorganized actin network throughout the culture period, with only limited structural evolution. In contrast, dynamic perfusion progressively promoted cytoskeletal alignment and interconnectivity, which became more pronounced from day 3 and further intensified on days 5 and 7.

At higher cell density (20 million cells/mL), static culture resulted in a densely packed but highly disordered network, consistent with locally dominated cell-mediated remodeling that persisted over time. Conversely, dynamically perfused constructs developed a dense, highly organized, and directionally aligned cytoskeletal structure, with alignment along the flow direction becoming increasingly evident from day 3 onward and reaching a well-defined anisotropic organization by day 7.

**Figure 6:**
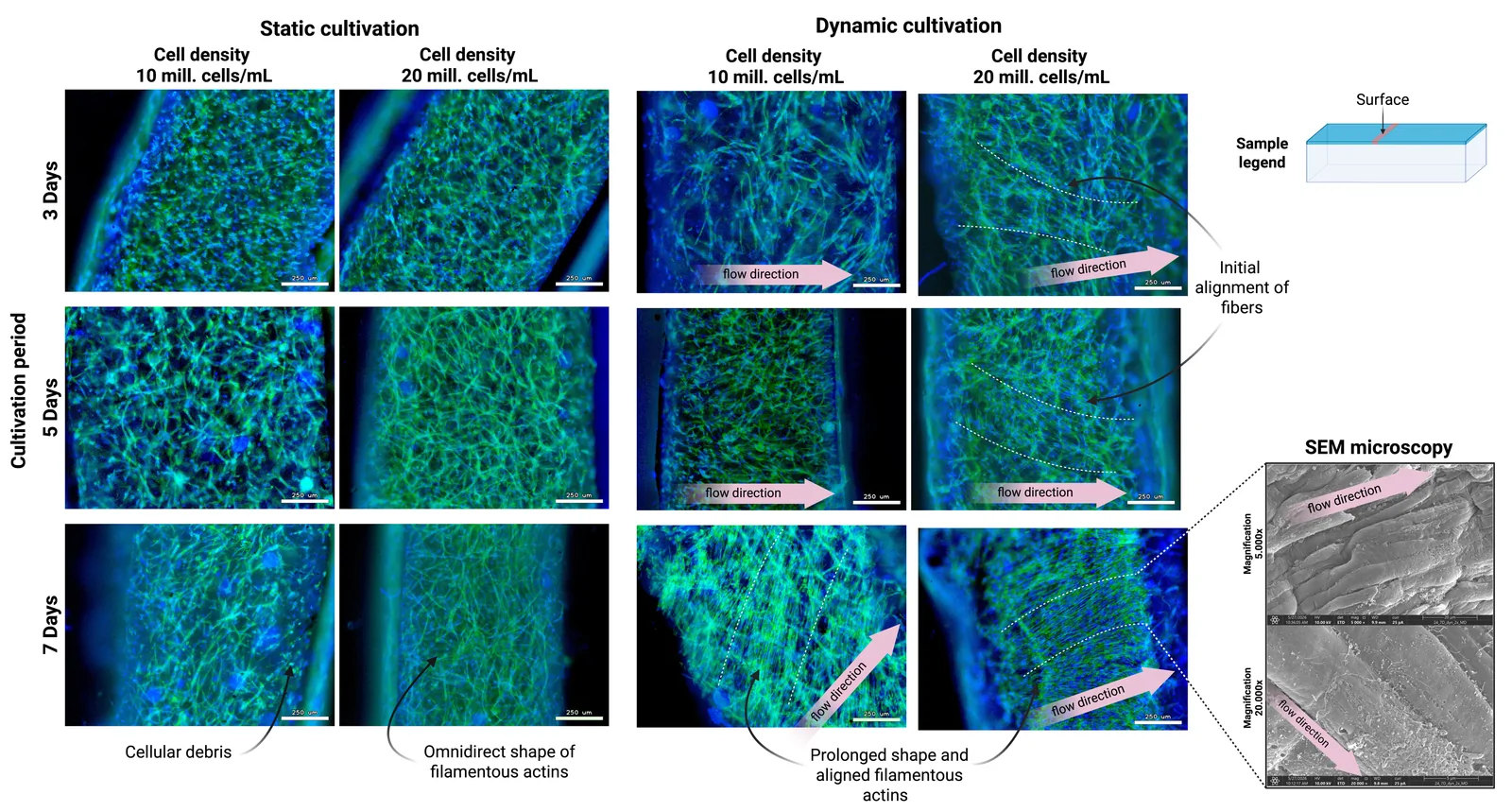
Top-view fluorescence images of nuclei (blue) and actin cytoskeleton (green) in constructs cultured under static and dynamic conditions at different cell densities (10 and 20 mil. cells/mL) over time (3–7 days). Dynamic perfusion promotes cytoskeletal alignment along the flow direction, as confirmed with SEM images (5000× and 20,000× magnification). Scale bars: 250 µm. Created in BioRender. Matějka, R. (2026) https://BioRender.com/dfvouyl.

Top-view (surface) fluorescence imaging (Figure 6) revealed a clear effect of culture conditions on cytoskeletal orientation. Under static culture, cells exhibited an isotropic and randomly organized actin network across all cultivation times and cell densities. In contrast, dynamically perfused constructs showed a progressive alignment of the cytoskeleton along the direction of flow, which became more pronounced with increasing cultivation time. This alignment was particularly evident at higher cell seeding density, where elongated filamentous structures followed a consistent orientation parallel to the perfusion direction. Scanning electron microscopy (SEM) of the construct surface further confirmed these observations. At 5000× magnification, cells under dynamic conditions displayed a flattened, elongated morphology with a clear orientation of cells along the flow axis. At higher magnification (20,000×), individual cells and associated extracellular matrix structures were visibly aligned parallel to the flow direction of culture media, consistent with the cytoskeletal reorganization observed in the fluorescence images. A detailed day-by-day comparison of cytoskeletal organization, including both surface slices and internal sections, is provided in Supplementary Materials part B (Figures S4–S7). In addition, a complete overview table of all samples from SEM microscopy is included in Supplementary Materials part C (Figure S8) for direct comparison.

In hydrogel-based 3D systems, medium perfusion and mass transfer critically influence cell distribution and phenotype [17]. The presented images indicate that remodeling outcomes are not determined solely by cell density but are strongly modulated by the culture environment over time. While higher cell density enhances the overall remodeling capacity, under static conditions, it can lead to disordered matrix reorganization due to excessive localized forces. Dynamic perfusion appears to mitigate this effect by promoting more uniform cellular activity and coordination, ultimately enabling controlled and structurally consistent remodeling, in agreement with previous studies demonstrating that perfusion enhances functional 3D tissue formation compared to static culture [17,18,19].

Cell-mediated compaction and applied flow further contribute to matrix reorganization and guide cell alignment, while the porous hydrogel structure allows cell migration, proliferation, and communication via soluble factors [17]. The progressive alignment of the cytoskeletal network along the direction of perfusion suggests that dynamic culture provides sustained directional cues that regulate cellular organization. This effect intensified over time and with increasing cell density, indicating that both external mechanical stimuli and collective cell behavior contribute to the development of anisotropic, organized structures within the constructs. In contrast, statically cultured samples retained a predominantly isotropic architecture throughout the cultivation period.

### 2.4. Geometry Changes and Remodeling Behavior Under Static and Dynamic Conditions

**Figure 7:**
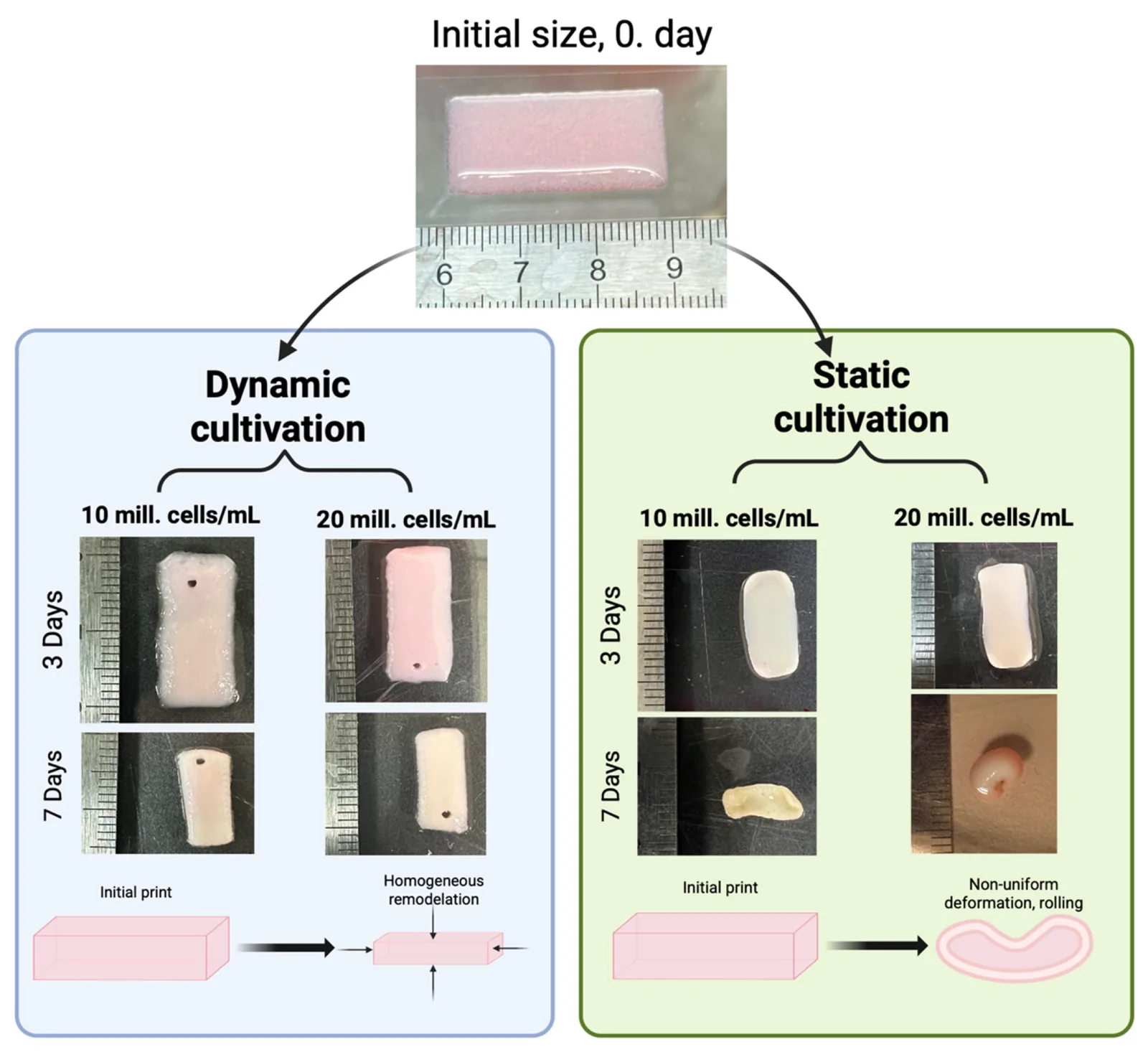
Remodeling behavior of constructs under dynamic and static culture. Dynamic samples show homogeneous contraction, while static samples exhibit non-uniform deformation and rolling. Created in BioRender. Matějka, R. (2026) https://BioRender.com/8xiddyl.

A clear qualitative difference in remodeling behavior was observed between static and dynamic cultivation conditions. While statically cultured constructs progressively lost their planar geometry and frequently rolled into tubular or irregular shapes over time, dynamically perfused samples retained their original rectangular form and exhibited a more uniform and isotropic reduction in size (Figure 7). This contrast indicates that dynamic culture promotes controlled and spatially homogeneous remodeling, whereas static conditions lead to non-uniform deformation driven by localized cell-mediated contraction.

**Figure 8:**
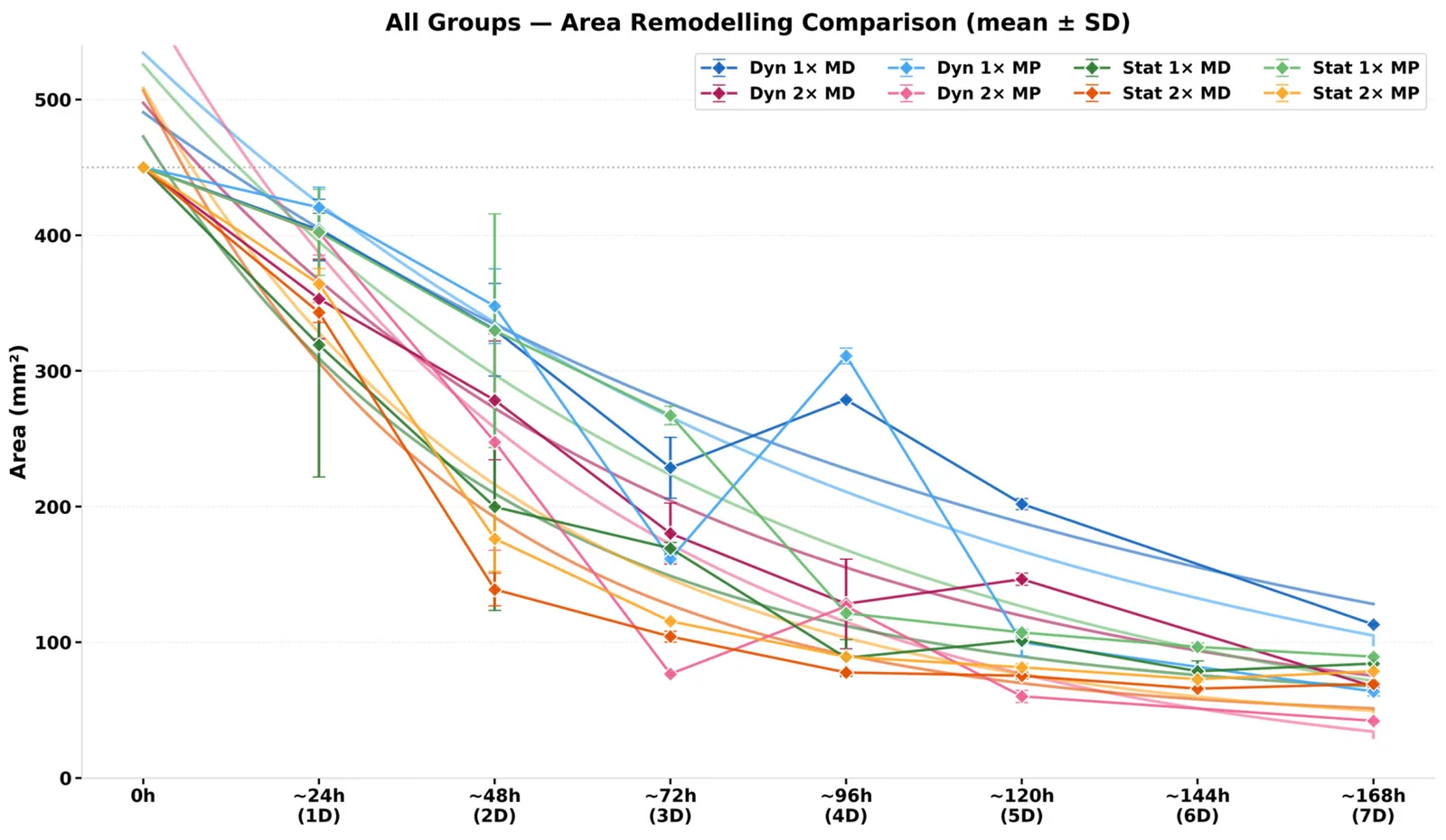
Overview of area remodeling across all eight experimental groups. Mean ± SD of projected surface area at each time point for all combinations of cultivation mode (dynamic/static), cell density (1× = 10 million cells/mL; 2× = 20 million cells/mL), and medium (MD/MP). Exponential decay fits are shown for each group.

All experimental groups exhibited progressive, time-dependent contraction over the 7-day cultivation period (Figure 8). Starting from an initial area of 450 mm^2^, all constructs underwent substantial size reduction. By day 7, final areas ranged from approximately 40 to 110 mm^2^, corresponding to a loss of ~75–90% of the initial surface area. The contraction followed a typical exponential trend, with the most rapid remodeling occurring during the first 72 h after bioprinting.

Despite differences in cultivation mode, medium, and cell density, all groups converged toward similarly reduced final dimensions. Variations between conditions were primarily reflected in the rate and timing of contraction rather than in the final extent of remodeling. Constructs with higher cell density and those cultured in MP medium generally exhibited faster contraction, whereas lower density and MD conditions showed slower remodeling trajectories. Overall, these findings indicate that collagen hydrogel remodeling follows a predictable cell-driven process, with experimental parameters modulating its kinetics rather than the underlying mechanism.

#### 2.4.1. Comparison of All Dynamic Groups

**Figure 9:**
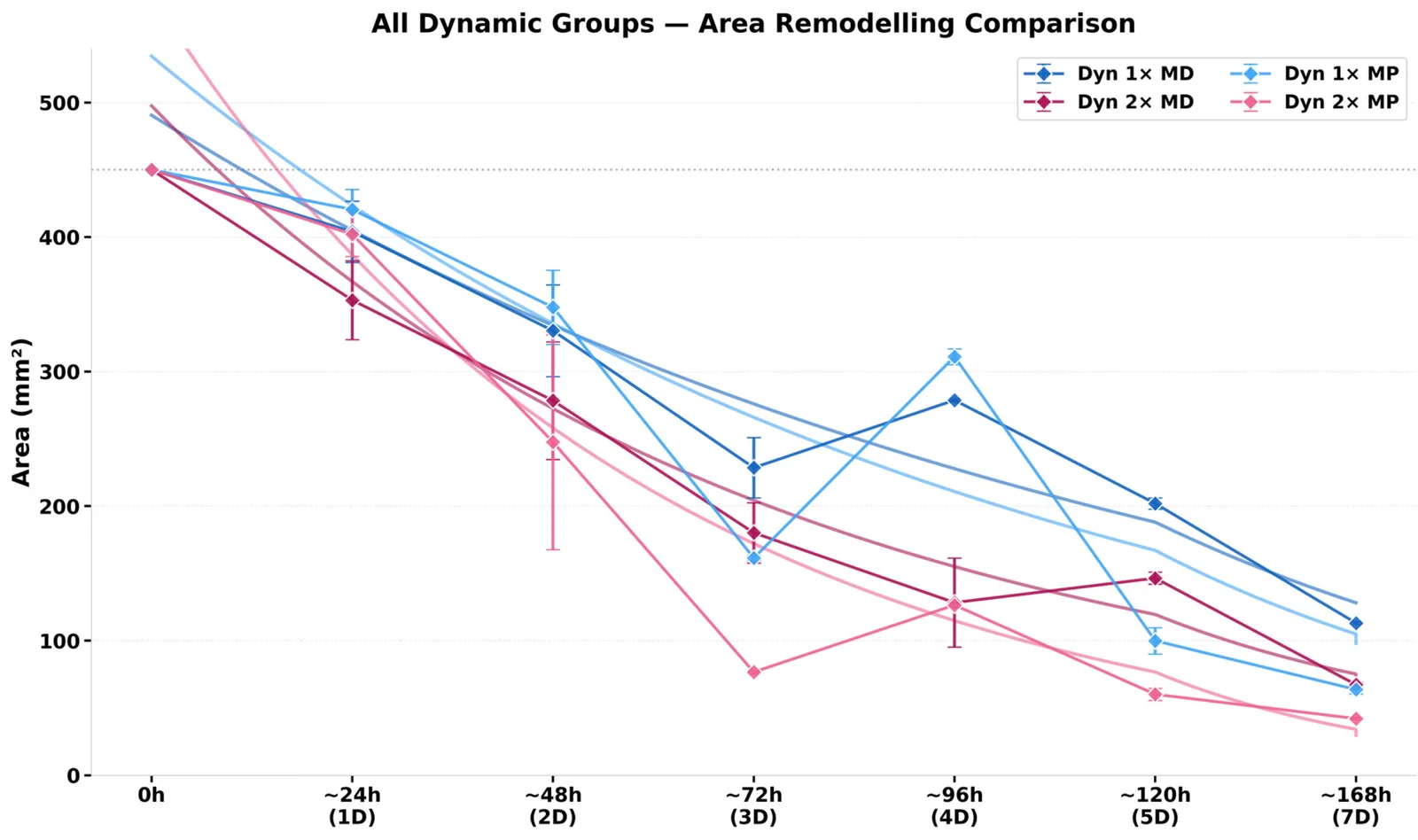
Comparison of remodeling kinetics across all four dynamic cultivation groups. Mean ± SD of projected surface area over time for constructs cultured dynamically at two cell densities (1× = 10 million cells/mL; 2× = 20 million cells/mL)) and in two media (MD, MP). Exponential decay fits are shown as solid lines.

When all four dynamic cultivation groups were evaluated together (Figure 9), a clear hierarchy of remodeling rates emerged. The 2× MP (proliferation culture medium with cell density 20 mil. cells/mL) group showed the fastest and most extensive contraction, followed by 2× MD (differentiation culture medium with cell density 20 mil. cells/mL), then 1× MP (proliferation culture medium with cell density 10 mil. cells/mL), while the 1× MD (differentiation culture medium with cell density 10 mil. cells/mL) group consistently exhibited the slowest remodeling trajectory. This ordering reflects the additive influence of cell density and medium composition under dynamic conditions. The separation between groups was most pronounced during the 48–120 h interval, when contraction rates differed substantially. After this period, all groups had already undergone major area reduction, and differences in absolute surface area became less distinct as they approached similarly low final values.

#### 2.4.2. Effect of Cell Density on Remodeling

**Figure 10:**
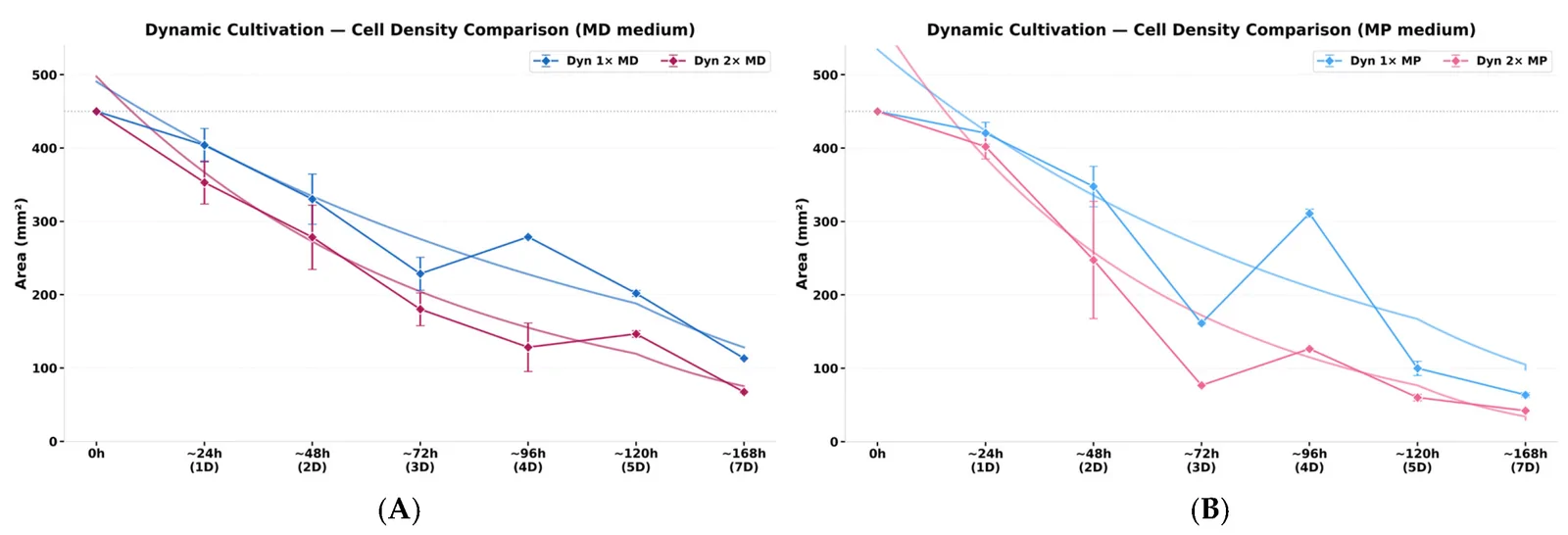
Effect of cell density on area remodeling under dynamic cultivation. (**A**) surface area reduction in constructs cultured in MD medium comparing 1× (10 million cells/mL) and 2× (20 million cells/mL) cell densities. (**B**) Corresponding remodeling profiles in MP medium.

Cell seeding density had a clear and consistent impact on remodeling across all conditions. Under dynamic cultivation, constructs with higher cell density (20 million cells/mL) exhibited faster and more pronounced contraction compared to those with lower density (10 million cells/mL) (Figure 10A). This difference emerged early during cultivation, with noticeable divergence already within the first 24 h, and continued to increase over time, resulting in substantially smaller final construct areas in the high-density groups.

A similar trend was observed in both media conditions, confirming that increased cell density accelerates matrix compaction independent of medium composition (Figure 10B). The accelerating effect of higher cell density aligns with the expected increase in cellular traction forces acting on the collagen network, which enhances matrix compaction and drives faster hydrogel remodeling.

#### 2.4.3. Effect of Cultivation Conditions—Dynamic vs. Static Conditions on Remodeling

The influence of cultivation type—dynamic cultivation in bioreactor vs. static culture—on remodeling was evident across all groups, although the magnitude of this effect varied depending on medium and cell density.

**Figure 11:**
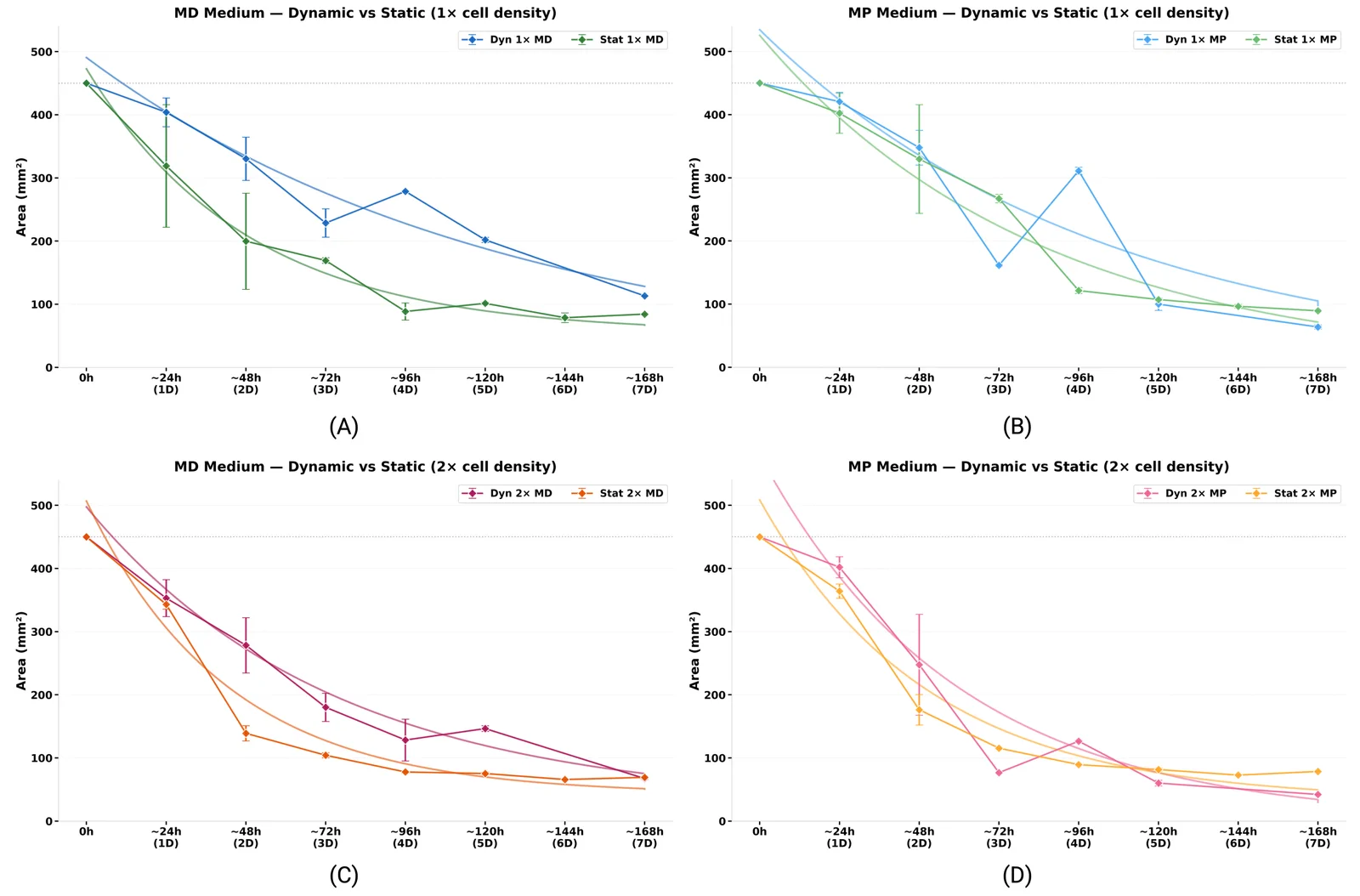
Effect of cultivation conditions (dynamic vs. static) on area remodeling of bioprinted collagen constructs. ((**A**)—top left) Remodeling profiles at 1× cell density (10 mil. cells/mL) in MD medium. ((**B**)—top right) Remodeling at 1× density in MP medium. ((**C**)—bottom left) Remodeling at 2× density in MD medium. ((**D**)—bottom right) Remodeling at 2× density (20 mil. cells/mL) in MP medium.

In MD medium at 1× density, dynamically cultivated samples showed a more gradual early phase remodeling trajectory, reaching 330.4 ± 34.1 mm^2^ at ~48 h, whereas static samples contracted more rapidly and with greater variability, decreasing to 199.8 ± 76.2 mm^2^ over the same period (Figure 11A). A similar pattern was observed at 2× density in MD medium: static constructs again contracted faster in the early phase (~48 h: 138.9 ± 12.0 mm^2^, 30.9% of initial), compared to 278.4 ± 43.8 mm^2^ (61.9% of initial) under dynamic cultivation (Figure 11C).

In MP medium, differences between dynamic and static conditions were less pronounced at the earliest time point (~24 h) but became increasingly apparent from ~48 h onward (Figure 11B, D). Static cultivation in MP medium also showed greater inter-sample variability, particularly during the early remodeling phase (~24–48 h), suggesting that the absence of flow-induced cues may lead to less homogeneous remodeling.

By the end of the cultivation period (~168 h), both dynamic and static groups converged toward similarly low final areas within each medium density combination. This indicates that while cultivation mode strongly influences the rate and early trajectory of remodeling, it has a more limited effect on the final contracted state of the constructs. This behavior aligns with expected differences in flow-mediated mechanical stimulation and nutrient transport between static and dynamic environments.

#### 2.4.4. Effect of Cultivation Medium—Proliferation or Differentiation on Remodeling

**Figure 12:**
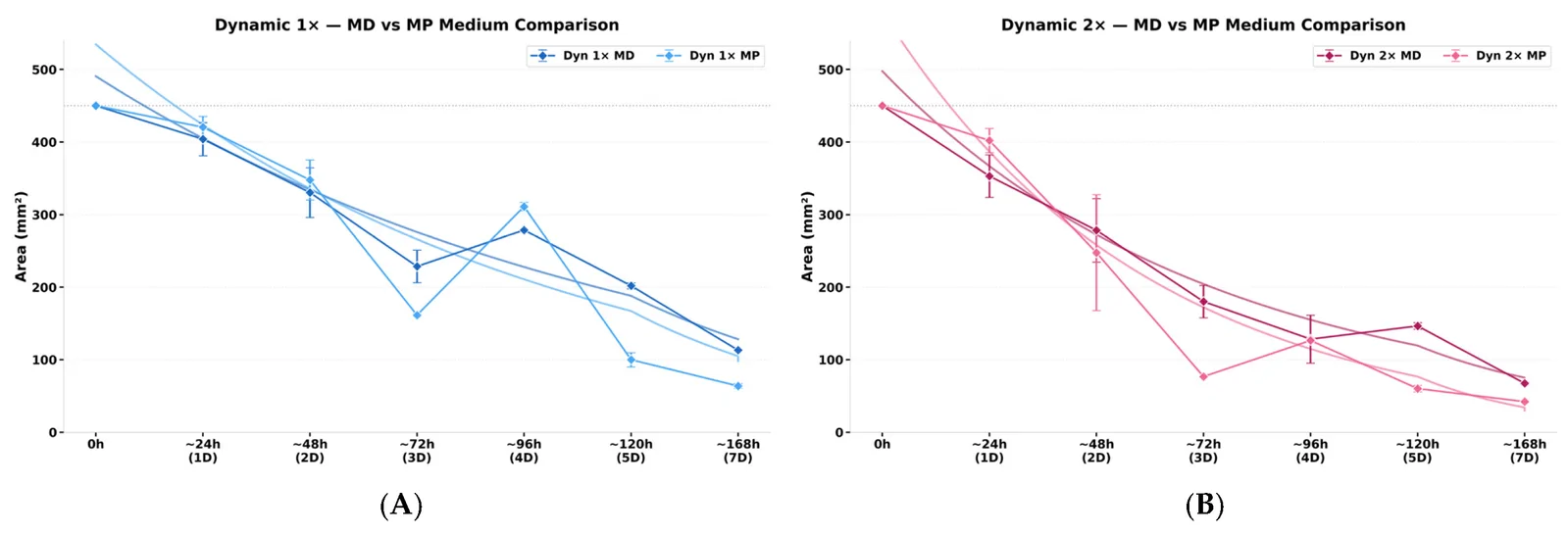
Effect of cultivation medium (MD vs. MP) on area remodeling under dynamic conditions. (**A**) Remodeling profiles at 1× cell density (10 mil. cells/mL. Mean ± SD of projected surface area for constructs cultured in MD (dark blue) and MP (light blue) media. Exponential decay fits are shown as solid lines. (**B**) Remodeling profiles at 2× cell density (20 mil. cells/mL). Mean ± SD for MD (red) and MP (pink) groups.

Culture medium significantly influenced remodeling kinetics, particularly during the intermediate phase of cultivation, as shown in Figure 12. Under dynamic conditions at low cell density, constructs in MP medium contracted more rapidly than those in MD, with clear differences emerging between 48 and 72 h. For example, by ~72 h, MP samples reached substantially smaller areas than MD constructs (≈161 mm^2^ vs. ≈229 mm^2^), despite showing similar sizes at earlier time points.

This effect was even more pronounced at higher cell density, where MP-cultured constructs exhibited markedly faster contraction, reaching less than half the area of their MD counterparts at ~72 h. These results indicate that medium composition strongly modulates the rate of remodeling, likely by influencing cell-mediated contractility and matrix reorganization.

These observations suggest that medium composition modulates the activity of embedded cells, potentially through differences in growth factor content, osmolarity, or other bioactive components that influence cell-mediated contractility and matrix remodeling within collagen hydrogels.

#### 2.4.5. Multifactorial Analysis of Remodeling and Summary

Based on the linear regression analysis (Table 1), the most significant factors affecting dimensional changes in area and tissue remodeling are the cultivation period, cell density, and cultivation method. In this context, static cultivation exerts a greater influence; however, it tends to have an undesirable impact, resulting in substrate warping as demonstrated in Figure 7. Additionally, there is a notable difference between proliferation and differentiation culture media. Proliferation media containing bFGF-2 support stem cell expansion but suppress differentiation and gene expression [20]. Mechanical evaluations also reveal differences between static and dynamic conditions, particularly in substrate elasticity and ductility, correlating with these results, described later.

Bioprinted collagen constructs exhibited a consistent, time-dependent contraction across all experimental conditions, reflecting active cell-mediated remodeling of the hydrogel matrix. The most rapid changes occurred during the early cultivation phase, indicating that initial remodeling dynamics play a dominant role in determining final construct geometry. Cell seeding density significantly accelerated the rate of contraction, while culture medium influenced the timing and progression of remodeling, with MP conditions promoting faster intermediate-stage compaction. Dynamic cultivation further resulted in more controlled and spatially uniform remodeling compared to static culture, which led to non-uniform deformation and rolling. Together, these findings demonstrate that remodeling behavior is governed by the interplay of cell density, culture environment, and transport conditions, primarily affecting the kinetics rather than the final extent of contraction.

Although substantial differences in remodeling kinetics were observed between culture conditions, media, and cell seeding densities, all groups ultimately converged toward similarly reduced dimensions. This behavior suggests that cell-mediated contraction is governed by a common equilibrium state determined by the balance between cellular traction forces and the mechanical resistance of the progressively compacted collagen matrix [21]. Under this framework, cell density, culture medium, and perfusion primarily influence the rate and trajectory of remodeling rather than the final extent of contraction. Higher cell densities generate stronger collective traction forces and accelerate matrix compaction, while culture conditions modulate cellular organization, cytoskeletal tension, and remodeling dynamics [21,22]. Similarly, dynamic perfusion promotes a more homogeneous distribution of nutrients and mechanical cues, thereby affecting the spatial pattern of remodeling without necessarily altering the eventual equilibrium state.

Importantly, similar final dimensions do not imply identical remodeling mechanisms. In the present study, constructs reached comparable levels of contraction through distinct remodeling pathways, as evidenced by pronounced differences in construct geometry, cytoskeletal organization, extracellular matrix architecture, and mechanical behavior. Dynamic cultivation preserved construct geometry and promoted more uniform remodeling, whereas static culture resulted in localized contraction and rolling despite reaching comparable final dimensions. Likewise, MP and MD media produced markedly different rates of contraction and matrix organization, although the final degree of compaction was similar. Together, these findings suggest that the final construct size is constrained by the mechanical limits of the remodeled collagen network, whereas culture conditions determine how that state is achieved and the structural and mechanical properties of the resulting tissue [22,23].

Remodeling of 3D hydrogels by cells significantly affects the final size and stability of the construct, with the most prominent manifestation being dimensional reduction driven by cell-generated forces [24]. It is a natural process, when contraction can facilitate construct handling and removal, but excessive or uncontrolled shrinkage may compromise tissue integrity, highlighting the need to balance scaffold degradation with cell-mediated matrix production for successful tissue engineering [24,25]. Cells, such as smooth muscle cells, exert traction forces on the hydrogel matrix, leading to gradual compaction of its porous network and a decrease in overall volume [24]. However, as our results have shown, factors other than cellular activity also influence the remodeling of hydrogel structures—dynamic perfusion, culture medium, and cell seeding density jointly govern the remodeling and maturation of bioprinted collagen constructs. Compared with static culture, perfusion promoted more uniform and predictable remodeling, preserved construct geometry, and supported a more organized cytoskeletal architecture with alignment along the flow direction. In contrast, static conditions led to uncontrolled deformation and loss of structural integrity.

Under static culture conditions, mass transport of oxygen, nutrients, and metabolic waste relies solely on passive diffusion, which is inherently limited to a depth of roughly 100–200 μm in dense, cell-laden hydrogels [26]. As a result, cells near the construct periphery experience a more favorable microenvironment than those in the core, generating radial gradients in nutrient and oxygen availability and in the accumulation of metabolic byproducts [27,28]. Such gradients are known to translate into spatial differences in cell viability, contractility, and matrix turnover, with peripheral regions typically showing more active remodeling than the core [27,29]. This spatial heterogeneity in cellular activity likely underlies the non-uniform compaction and deformation observed in statically cultured constructs, as unevenly distributed traction forces create local imbalances in internal mechanical tension across the construct.

Dynamic culture addresses this limitation through two complementary mechanisms. First, continuous medium flow improves the delivery of nutrients and oxygen while also enhancing clearance of metabolic waste, reducing periphery-to-core gradients, and supporting more spatially uniform cell viability and metabolic activity [29,30].

Second, fluid flow exposes embedded cells to shear stress, which is transduced into intracellular signaling via integrin- and cytoskeleton-mediated mechanotransduction pathways [31] and can modulate cell contractility and matrix gene expression [32]. The combined effect of improved mass transport and shear-induced mechanical stimulation likely promotes a more spatially homogeneous pattern of matrix degradation and deposition, consistent with the more uniform remodeling we observed under dynamic conditions.

These findings suggest that remodeling can be strategically harnessed to tune both the mechanical properties and final architecture of the constructs, with external factors, such as flow and intrinsic parameters, such as cell density, serving as key regulators of construct maturation. The dimensional changes caused by cell remodeling can be controlled in vitro by mechanical fixation, for example, by anchoring the construct to prevent shortening, or by modifying matrix composition through the addition of more stable fibers, such as collagen, to increase mechanical resistance [8,24].

### 2.5. Mechanical Properties of Static and Perfusion-Cultured Constructs

The remodeling of cell-laden hydrogels is a dynamic process that fundamentally transforms the mechanical properties of the construct as it evolves from an initial scaffold into a tissue-like structure [20]. The mechanical properties of the remodeled tissue constructs were evaluated under several experimental conditions: across predefined cultivation periods (0, 3, and 7 days), between static and dynamic cultivation regimes, and between proliferation (MP) and differentiation (MD) media. In all cases, the elastic modulus was determined from the linear region of the mean stress–strain curves, with dashed lines indicating the corresponding linear fits. The min–max envelopes reflect inter-sample variability within each group. Each figure includes macroscopic photographs of the constructs at rest, at the onset of stretching, and at maximum deformation prior to rupture, alongside SEM cross-sectional images illustrating the ultrastructural changes associated with each condition.

**Figure 13:**
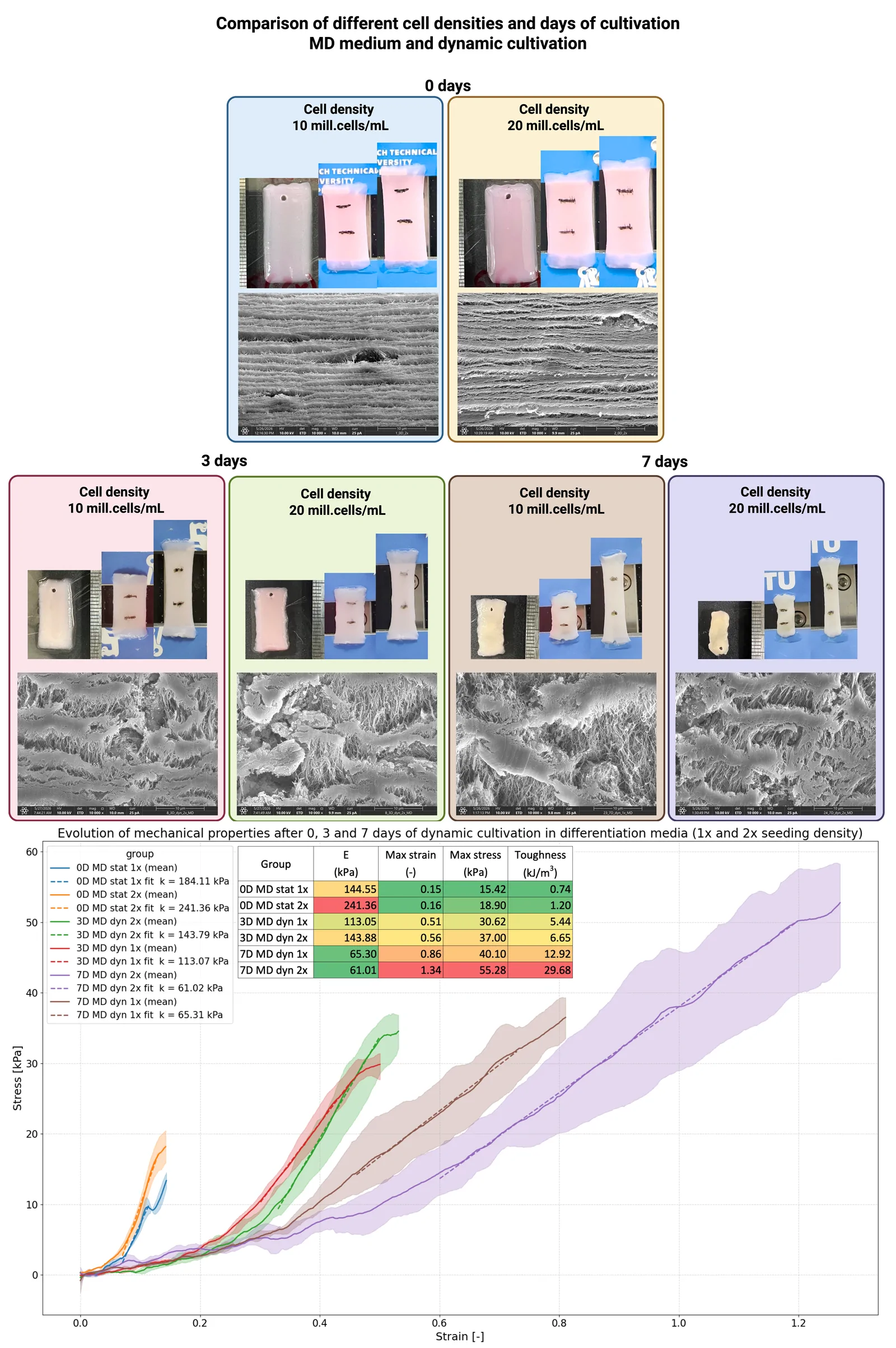
Mechanical characterization and structural analysis of tissue constructs during dynamic cultivation in differentiation medium over time. Mean stress–strain curves (solid lines) with min–max envelopes (shaded areas) and linear fits (dashed lines, elastic modulus k in kPa) are shown for constructs cultured for 0, 3, and 7 days at both cell seeding densities (10 and 20 mil. cells/mL). Macroscopic images illustrate the extent of construct deformation at the onset of stretching and at maximum strain prior to rupture. SEM cross-sections show the progressive ultrastructural remodeling of the scaffold interior, from the native striated fiber architecture at day 0 to the development of thick, bundled actin-like fibers and dense ECM deposition by day 7. The summary table provides the elastic modulus (E), maximum strain, maximum stress, and toughness for each group. Created in BioRender. Matějka, R. (2026) https://BioRender.com/ps245kh.

The stress–strain curves obtained from dynamically cultivated samples in differentiation media reveal a clear and consistent trend with increasing cultivation duration (Figure 13). At day 0, constructs exhibited the highest stiffness, with elastic moduli of 144.55 kPa (cell density 10 mil. cells/mL) and 241.36 kPa (cell density 20 mil. cells/mL) and failed at very low strains (ε ≈ 0.15–0.16), reflecting the brittle, largely acellular character of the initial scaffold. Macroscopically, day 0 constructs appeared uniform, consistent with freshly prepared cell-laden hydrogels, and ruptured abruptly with minimal deformation. By day 3, the elastic modulus decreased substantially to 113.05 kPa (10 mil. cells/mL) and 143.88 kPa (20 mil. cells/mL), while the maximum strain increased to ε ≈ 0.51–0.56, indicating early onset of remodeling. By day 7, the modulus further decreased to 65.30 kPa (10 mil. cells/mL) and 61.01 kPa (20 mil. cells/mL), while maximum strain extended to ε ≈ 0.86–1.34, demonstrating a markedly more compliant and ductile character. Macroscopically, day 7 constructs sustained substantially greater deformation before failure compared to day 0, which ruptured abruptly with minimal elongation. SEM imaging of the cross-sections corroborated these findings: at day 0, the scaffold showed a layered, striated fibrous architecture with rounded, undifferentiated cells and no visible ECM remodeling. At day 3, the internal structure became more disrupted, with emerging cell-derived fibers beginning to integrate with the scaffold. By day 7, thick, prominently bundled actin-like fibers were clearly visible alongside regions of dense ECM deposition, likely reflecting collagen accumulation, collectively indicative of advanced matrix remodeling and structural reorganization. Higher initial cell seeding density (20 million cells/mL) resulted in greater maximum stress at day 7 (55.28 kPa vs. 40.10 kPa), suggesting that denser cell populations drive more extensive matrix production and load-bearing capacity development. This progressive remodeling was also reflected in the toughness (work to failure), which increased markedly with cultivation time, from ≈ 0.74–1.2 kJ/m^3^ at day 0 to ≈ 12.92–29.68 kJ/m^3^ at day 7, confirming a substantial gain in the energy absorbed before failure despite the decrease in elastic modulus.

**Figure 14:**
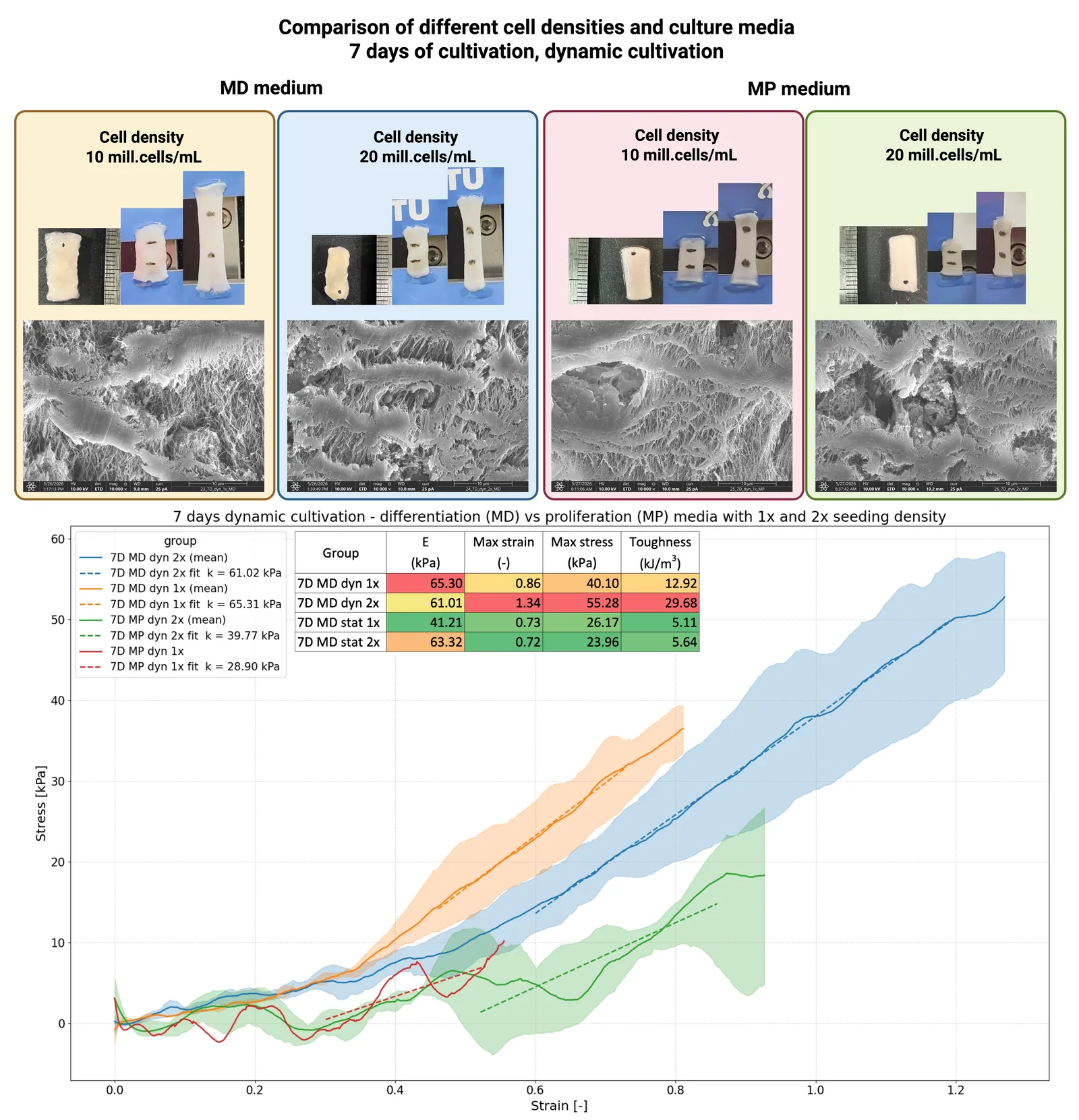
Mechanical characterization and structural analysis of tissue constructs under static and dynamic cultivation in differentiation medium after 7 days. Mean stress–strain curves (solid lines) with min–max envelopes (shaded areas) and linear fits (dashed lines, elastic modulus k in kPa) are shown for statically and dynamically cultured samples at both cell seeding densities (10 and 20 million cells/mL). Macroscopic images illustrate the extent of construct deformation at the onset of stretching and at maximum strain prior to rupture. SEM cross-sections reveal differences in internal fiber organization, with dynamically cultivated constructs showing thicker, more bundled fiber structures and denser ECM compared to the more heterogeneous architecture of static constructs. The summary table provides the elastic modulus (E), maximum strain, maximum stress, and toughness for each group. Created in BioRender. Matějka, R. (2026) https://BioRender.com/hap3k11.

The comparison of static and dynamic cultivation in MD medium after 7 days (Figure 14) reveals a moderate but meaningful difference in mechanical behavior. Dynamically cultivated constructs exhibited elastic moduli of 65.30 and 61.01 kPa (10 and 20 mil. cells/mL cell density, respectively), while sustaining considerably greater deformation, with maximum strains reaching ε ≈ 0.86–1.34, compared to ε ≈ 0.72–0.73 under static conditions. Macroscopically, dynamic constructs demonstrated notably greater elongation at failure, whereas static constructs exhibited more abrupt rupture at lower deformation levels. SEM cross-sections reflected these differences in internal organization: dynamically cultivated constructs displayed thicker, more bundled fiber structures with a denser and more continuous ECM, whereas static constructs showed a more heterogeneous internal architecture with less directionally organized fibers and more irregular structural voids. This behavior may be attributed to the mechanical stimulation inherent to dynamic cultivation, which is known to promote cell alignment, cytoskeletal organization, and extracellular matrix remodeling, favoring extensibility over stiffness. The broader variability envelope observed in the static group at intermediate strain levels further suggests less uniform structural development in the absence of flow-induced mechanical stimulation. This was also reflected in the toughness, which was markedly higher under dynamic than static cultivation (12.92 and 29.68 kJ/m^3^ dynamically vs. 5.11 and 5.64 kJ/m^3^ statically, at 10 and 20 million cells/mL), confirming a substantially greater capacity to absorb energy before failure under perfusion.

**Figure 15:**
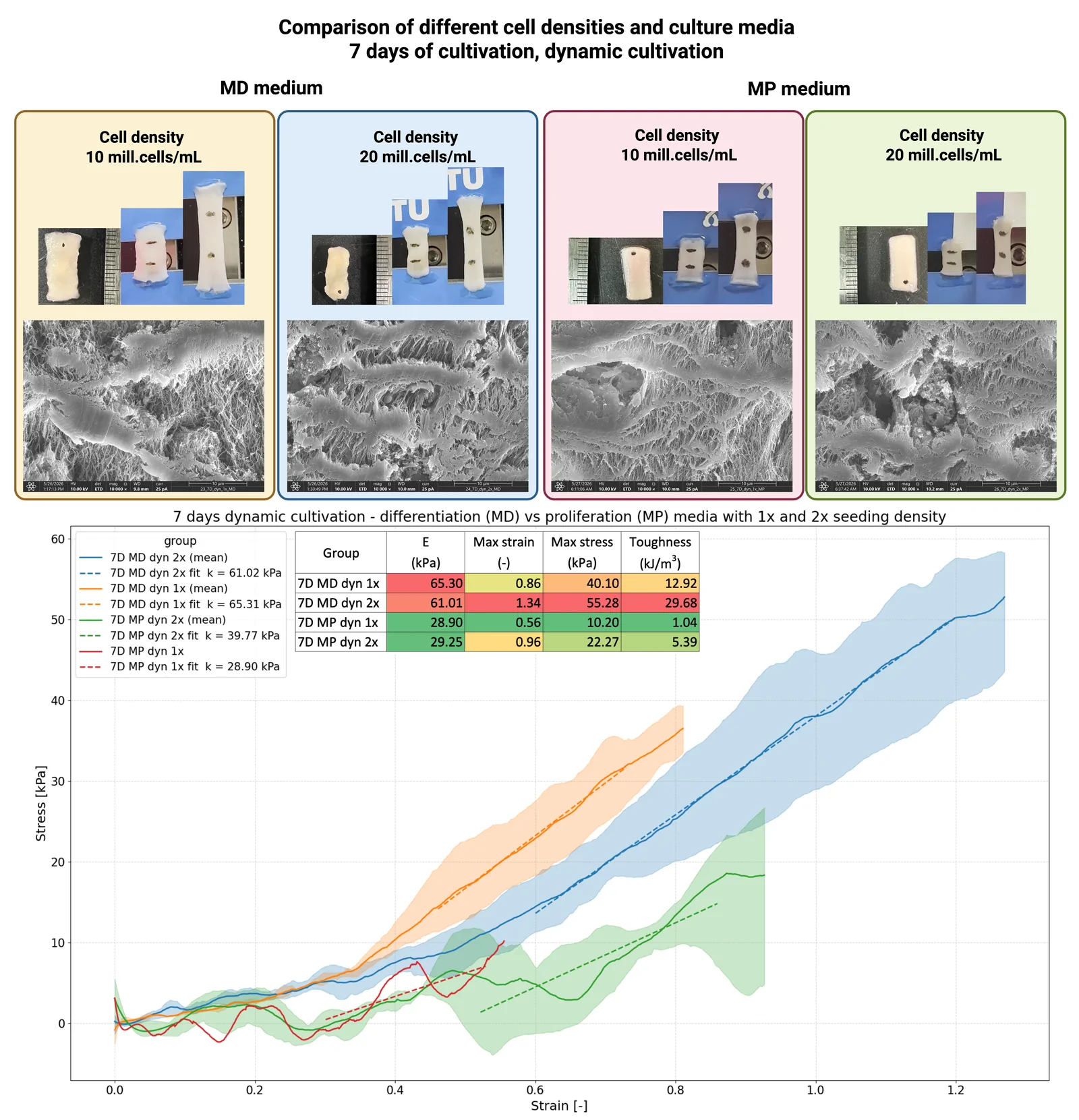
Mechanical characterization and structural analysis of tissue constructs cultured in proliferation (MP) and differentiation (MD) media after 7 days under dynamic conditions. Mean stress–strain curves (solid lines) with min–max envelopes (shaded areas) and linear fits (dashed lines, elastic modulus k in kPa) are shown for both media at both cell seeding densities (10 and 20 mil. cells/mL). Macroscopic images illustrate the extent of construct deformation at the onset of stretching and at maximum strain prior to rupture. SEM cross-sections highlight the striking ultrastructural differences between conditions. The summary table provides the elastic modulus (E), maximum strain, maximum stress, and toughness for each group. Created in BioRender. Matějka, R. (2026) https://BioRender.com/kbd3oae.

The comparison between MD and MP media after 7 days of dynamic cultivation (Figure 15) revealed the most substantial differences in both mechanical performance and ultrastructural organization among all tested conditions. MD constructs exhibited elastic moduli of 65.30 kPa (10 mil. cells/mL) and 61.01 kPa (20 mil. cells/mL), more than twice those of the MP group (28.90 and 29.25 kPa, respectively). MD constructs also sustained significantly higher stress levels, reaching approximately 40.10 and 55.28 kPa at maximum strain, compared to only 10.20 and 22.27 kPa in the MP group. The stress–strain curves of MD constructs were smooth and monotonically increasing, while MP curves displayed notable irregularities and fluctuations, reflecting a less structurally organized material. Macroscopically, MD constructs sustained markedly greater elongation before failure, while MP constructs ruptured at considerably lower strains and appeared more fragile during handling. SEM cross-sections strikingly illustrated the underlying structural basis of these differences: MD constructs featured thick, well-developed, bundled fiber structures with substantial dense ECM regions, consistent with robust actin stress fiber formation and collagen deposition under differentiation conditions. MP constructs, by contrast, showed considerably thinner and more loosely arranged fibers with less coherent ECM organization, suggesting that proliferative conditions support cell expansion but do not drive the level of cytoskeletal maturation and matrix production necessary for robust mechanical performance. These differences were equally pronounced in the toughness, which was several-fold higher for MD than MP constructs (12.92 and 29.68 kJ/m^3^ vs. 1.04 and 5.39 kJ/m^3^, at 10 and 20 million cells/mL), consistent with the higher failure stress and strain and the more developed fibrillar architecture of the differentiation-medium group.

**Figure 16:**
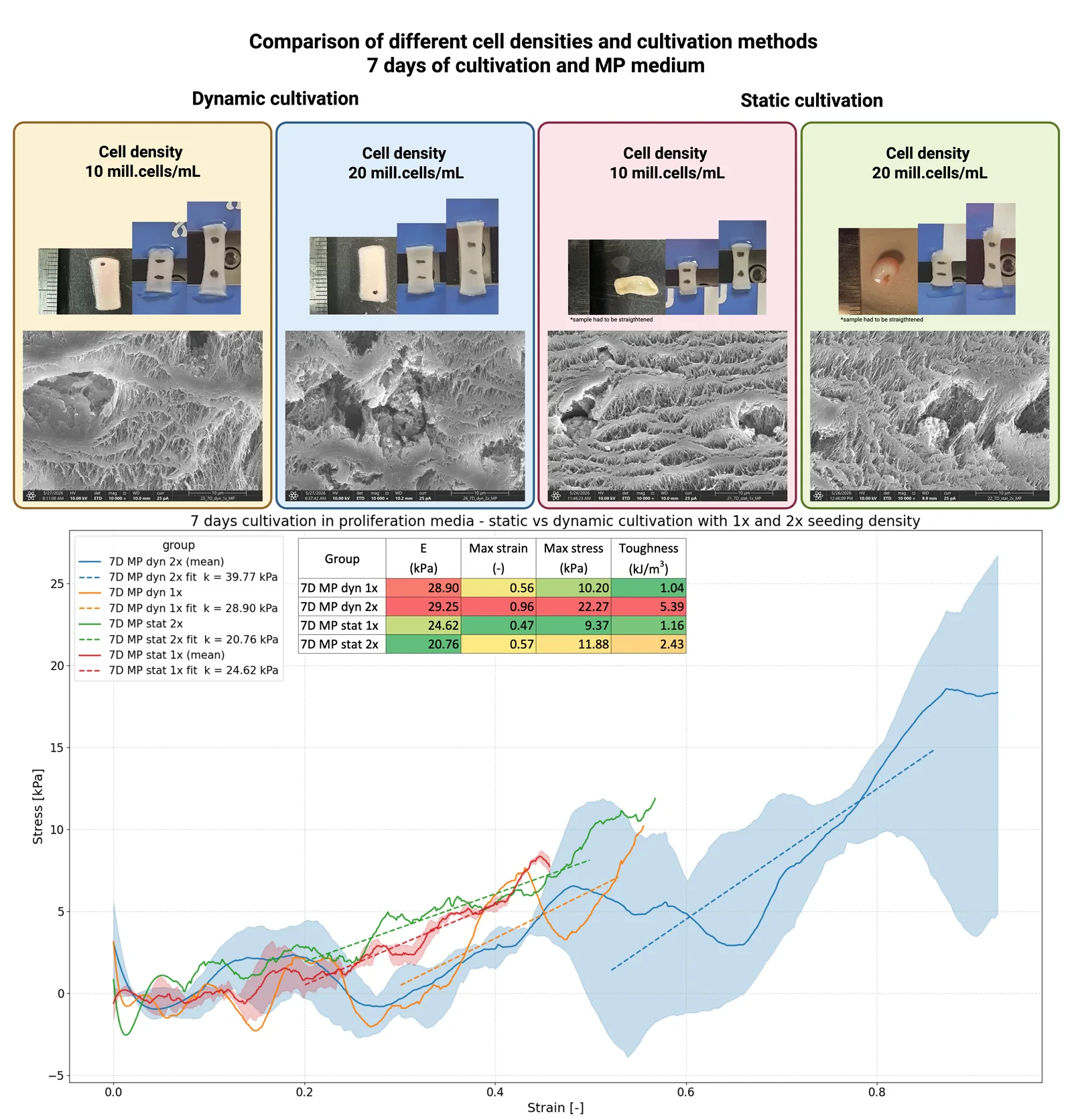
Mechanical characterization and structural analysis of tissue constructs under static and dynamic cultivation in proliferation medium after 7 days. Mean stress–strain curves (solid lines) with min–max envelopes (shaded areas) and linear fits (dashed lines, elastic modulus k in kPa) are shown for statically and dynamically cultured samples at both cell seeding densities (10 and 20 mil. cells/mL). Macroscopic images illustrate the extent of construct deformation at the onset of stretching and at maximum strain prior to rupture, noting that static constructs at higher cell density required straightening prior to testing due to spontaneous contractile deformation during culture. SEM cross-sections show thin, loosely organized fiber networks with irregular ECM deposits across all groups. The summary table provides the elastic modulus (E), maximum strain, maximum stress, and toughness for each group. Created in BioRender. Matějka, R. (2026) https://BioRender.com/dckozgr.

The comparison of static and dynamic cultivation in MP medium after 7 days (Figure 16) showed comparatively modest differences relative to the MD medium groups. Elastic moduli were similar across conditions, ranging from 20.76 kPa (static, 20 mil. cells/mL) to 29.25 kPa (dynamic, 20 mil. cells/mL), and maximum strains were largely comparable (ε ≈ 0.47–0.96). Notably, static MP constructs at higher cell density required straightening prior to testing, indicating spontaneous contractile deformation during culture. Macroscopically, the extent of elongation at failure was limited across all MP groups relative to MD, consistent with the lower overall ductility of proliferation medium constructs. SEM imaging showed that both static and dynamic MP constructs contained thin, loosely organized fibers with irregular ECM deposits, though static constructs at higher density displayed somewhat more developed wavy fiber bundles, potentially reflecting passive compaction-driven fiber organization in the absence of perfusion. Together, these findings highlight that culture medium composition was the dominant factor governing both the ultrastructural development and mechanical maturation of the constructs, with dynamic cultivation providing a secondary but meaningful contribution, particularly under differentiation conditions. Toughness remained low across all MP groups (1.04–5.39 kJ/m^3^), with only minor differences between static and dynamic conditions, further underscoring the limited mechanical maturation achieved in proliferation medium.

Linear regression models also show these trends for Young’s modulus, maximal stress, and strain. Cultivation factor estimates are listed in Table 2 (Young’s modulus), Table 3 (maximal strain), Table 4 (maximal stress), and Table 5 (Toughness).

The reduction in Young’s modulus is primarily influenced by the proliferation medium, which affects the integrin complex and cell–substrate integration [20], and develops over days of cultivation. In contrast, higher cell density and dynamic cultivation generally lead to an increase in modulus.

Material elasticity and maximum stiffness are mainly affected by the differentiation medium, dynamic cultivation, and cell density. SEM and confocal images show that dynamic cultivation with differentiation media produces thick actin fibers bundled with remodeled collagen substrate.

Construct toughness followed the same trends, with cultivation time and cell density increasing it, while proliferation medium and static cultivation significantly reduced it (Table 5).

Throughout this work, we use the term mechanical maturation to denote the functional transition of the construct from a passive, brittle, quasi-linear cast-collagen scaffold to a cell-remodeled, tissue-like material exhibiting native-type nonlinear behavior—specifically, a reduced low-strain modulus (increased compliance), a markedly increased strain at failure and toughness, a J-shaped strain-stiffening response, and a shift in load-bearing from the as-fabricated dense collagen to an organized cell-synthesized fibrillar network. Importantly, maturation in this sense does not denote an increase in stiffness or a simple gain in mechanical strength. This distinction is essential to interpreting the data. At day 0, the material is a dense, laminar, compacted-collagen sheet (Figure 13 and Figure S8) that behaves as a brittle continuous solid: it exhibits a high elastic modulus (144–241 kPa) but fails at very low strain (0.15–0.16) and low ultimate stress (15–19 kPa). After 7 days of dynamic cultivation in MD medium, SEM images show that this dense lamellar architecture is largely replaced by an open, interconnected fibrillar network, with only residual regions of the original cast collagen (Figure 13 and Figure S8). Mechanically, this network is compliant at low strain, deforming by fiber uncrimping, reorientation, and sliding, and stiffens progressively at higher strain as fibers are recruited and aligned to the loading axis, producing the characteristic J-shaped stress–strain response of native collagenous soft tissue [33]. Consequently, the day-7 constructs show a lower initial modulus (61–65 kPa) yet a 5.7–8.4-fold higher strain at failure (0.86–1.34) and a 2.6–2.9-fold higher ultimate stress (40–55 kPa) than at day 0. Critically, this behavior is not a conventional strength-for-ductility trade-off. The construct simultaneously gained extensibility, ultimate strength, and toughness while relinquishing only low-strain stiffness. Toughness, quantified as the work to failure (area under the stress–strain curve), increased from ≈ 0.74–1.2 kJ/m^3^ at day 0 to ≈ 12.92–29.68 kJ/m^3^ at day 7 (MD medium, dynamic cultivation), an approximately 15–25-fold increase. Because the cultivated constructs are strongly nonlinear, the material is compliant in the low-strain toe region and considerably stiffer at high strain; the single linear-fit modulus therefore averages these two regimes and should not be read as uniform softening. For a soft-tissue or vascular-graft target, the reduced low-strain modulus is functionally advantageous, moving the construct away from the stiffness and compliance mismatch associated with brittle scaffolds and toward the compliant, strain-stiffening, high-toughness behavior of native tissue [34]. We therefore interpret the day-0-to-day-7 evolution as maturation in this functional, tissue-mimetic sense: the emergence of native-type mechanics driven by cellular remodeling of the matrix, rather than as an increase in mechanical strength.

During the process of remodeling, cells actively degrade the original matrix while simultaneously synthesizing and depositing their own extracellular matrix, leading to a gradual replacement of the initial material with a biologically derived network [25]. In parallel, cell-generated traction forces induce compaction of the hydrogel, increasing its density and internal tension, which contributes to enhanced mechanical integrity despite an overall reduction in size [25]. This remodeling may be further reinforced by biochemical feedback mechanisms associated with matrix degradation, which can stimulate cell activity and additional matrix production. Moreover, external stimuli, such as dynamic culture and mechanical loading, can further promote matrix reorganization and cellular alignment, thereby supporting the development of more functionally relevant mechanical properties [8]. Together, these processes contribute to the transition from a soft, low-strength hydrogel to a mechanically stable and handleable tissue-like construct [20]. The trends observed in our data are consistent with progressive tissue remodeling occurring during cultivation, including changes in scaffold porosity, cell orientation, fiber organization, and overall material homogeneity. The transition from a stiffer, more brittle response toward a more compliant and ductile behavior is a hallmark of maturing tissue constructs undergoing structural reorganization [8]. Mechanical analysis revealed a time-dependent transition from a stiff and brittle response at early stages to a more compliant and ductile behavior at later time points, indicating progressive matrix reorganization. Despite decreasing stiffness, constructs maintained or improved load-bearing capacity at higher strains. Dynamic culture increased extensibility, whereas static cultivation resulted in stiffer but less deformable constructs. Among all tested parameters (overview of all measured mechanical data shown in Supplementary Materials part D), culture medium had the strongest impact on mechanical performance, with differentiation medium promoting the development of a more mechanically robust and organized extracellular matrix compared to proliferation medium, which, while supporting cell growth, resulted in constructs with less developed load-bearing structures, highlighting that the choice of medium represents a key parameter in the design of tissue engineering protocols aimed at achieving targeted mechanical properties.

## 3. Conclusions

This study demonstrates that dynamic perfusion fundamentally alters the remodeling behavior and mechanical maturation of bioprinted collagen constructs. Under dynamic conditions, constructs exhibited more uniform and spatially controlled contraction, while preserving their original geometry, in contrast to static culture, which led to non-uniform deformation and rolling. Across all experimental groups, remodeling followed a consistent time-dependent trend with rapid contraction during the early cultivation phase, after which all constructs converged toward similarly reduced final dimensions, indicating that culture conditions primarily modulate remodeling kinetics rather than the final extent of contraction.

The remodeling process was strongly influenced by intrinsic and extrinsic parameters. Increasing cell seeding density accelerated matrix compaction and led to faster contraction, while culture medium composition significantly affected remodeling dynamics, particularly during the intermediate phase. Constructs cultured in proliferation medium showed faster contraction, whereas differentiation conditions resulted in more controlled remodeling and structurally coherent constructs. Dynamic perfusion further contributed to homogeneous remodeling and promoted a more organized cytoskeletal architecture, including directional alignment along the flow direction.

Mechanical analysis revealed a progressive transition from a stiff and brittle response at early stages to a more compliant and ductile behavior during cultivation, accompanied by improved strain tolerance while maintaining load-bearing capacity at higher deformation. Although dynamically cultured constructs exhibited a lower elastic modulus compared to static samples, they demonstrated enhanced extensibility and more consistent mechanical responses. The strongest effect on mechanical performance was observed for culture medium, where differentiation conditions yielded significantly more stable and homogeneous stress–strain behavior than proliferation conditions.

These findings indicate that remodeling is not merely a passive consequence of cell activity, but a controllable process governed by the interplay of cell-mediated forces, matrix degradation and synthesis, transport conditions, and biochemical signals. The observed coupling between cytoskeletal organization, matrix remodeling, and mechanical properties highlights the importance of coordinated culture conditions for achieving predictable construct maturation.

However, several limitations should be acknowledged. Smooth muscle-like differentiation was evaluated using α-SMA, calponin, and cytoskeletal organization; although the observed marker expression and pronounced cell alignment under dynamic cultivation suggest acquisition of a contractile, smooth muscle-associated phenotype, α-SMA and calponin are not specific to mature smooth muscle cells. Our findings therefore indicate early smooth muscle-like differentiation rather than definitive lineage commitment, and confirmation using late-stage markers and functional contractility assays is planned in a follow-up study. The relatively short cultivation period (7 days) captured the rapid contraction phase and the early stages of mechanical maturation but is unlikely to reflect long-term remodeling. Contraction had largely plateaued by day 7, whereas the mechanical properties were still evolving, indicating that the constructs had not yet reached a stable steady state. Key maturation processes relevant to load-bearing tissues typically unfold over weeks rather than days, so the longer-term stability of the observed mechanical state remains to be established. While the planar geometry used here is directly applicable to patch-type constructs, translation to tubular vascular grafts would require reproducing wall curvature and circumferential fiber alignment. In addition, the study relied on a single stromal cell source in monoculture, without the endothelial or other vascular cell types present in native tissue. Despite these constraints, the study provides a controlled framework for identifying key factors that govern remodeling and mechanical development in collagen-based systems. Future work should focus on extending cultivation time, exploring a broader range of perfusion and cell density conditions, and incorporating more complex multicellular and geometrically relevant models to further enhance physiological relevance and functional performance.

## 4. Materials and Methods

### 4.1. Growth Curve Analysis and Assessment of Culture Media in 2D Culture

Growth curve analysis was performed using 24-well glass-bottom plates (Corning, NY, USA) coated with gelatin. Briefly, a 1% (*w*/*v*) gelatin solution was added to each well and incubated for 2 h at 37 °C under 5% CO_2_. After incubation, the excess solution was aspirated, and the wells were allowed to dry under sterile conditions in a laminar flow hood prior to cell seeding. Wharton’s jelly-derived stromal cells were seeded at a density of 10,000 cells/cm^2^ and cultured in four experimental media formulations:

• Proliferative DMEM-based medium (DP): DMEM:F12 (1:1) (Dulbecco’s Modified Eagle Medium/Ham’s F-12; Gibco, Grand Island, NY, USA) supplemented with 2.438 g/L sodium bicarbonate (Sigma-Aldrich, Merck, St. Louis, MO, USA), 10% fetal bovine serum (FBS), and 10 ng/mL FGF2 (PeproTech, Cranbury, NJ, USA);
• Differentiation DMEM-based medium (DD): DMEM:F12 (1:1) supplemented with 10% FBS, 50 µg/mL ascorbic acid (Penta, Prague, Czech Republic), 2.5 ng/mL BMP4 (Abcam, Cambridge, UK), and 2.5 ng/mL TGF-β1 (PeproTech, Cranbury, NJ, USA);
• Proliferative MCDB-based medium (MP): MCDB131 supplemented with 1.18 g/L sodium bicarbonate (Sigma-Aldrich, Merck, St. Louis, MO, USA), 10% FBS, and 10 ng/mL FGF2;
• Differentiation MCDB-based medium (MD): MCDB131 supplemented with 10% FBS, 50 µg/mL ascorbic acid, 2.5 ng/mL BMP4, and 2.5 ng/mL TGF-β1.

Culture medium was replaced daily. Cells were cultured for 1, 2, 3, 4, 5, and 7 days, after which samples were fixed in 70% (*v*/*v*) ethanol for approximately 5 min and stained with DAPI to visualize cell nuclei.

To further characterize the effect of the four media on cell behavior, WJCs were seeded at cell densities of 10,000, 20,000, and 40,000 cells/cm^2^ in gelatin-coated 24-well plates and cultured for 1, 3, and 5 days, with daily medium exchange. At each time point, cellular metabolic activity was quantified using the AlamarBlue assay (Thermo Fisher Scientific, Waltham, MA, USA) according to the manufacturer’s instructions. Following metabolic activity analysis, samples were fixed with 4% (*w*/*v*) paraformaldehyde (PFA in PBS; Thermo Fisher Scientific, Waltham, MA, USA) and stained with DAPI for nuclei visualization, phalloidin for F-actin labeling (ReadyProbes™ F-actin Phalloidin conjugates, Invitrogen, ThermoFisher Scientific, Waltham, MA, USA), and α-smooth muscle actin (α-SMA; Abcam, Cambridge, UK) or calponin (Calponin-1, Invitrogen, ThermoFisher Scientific, Waltham, MA, USA) to assess cell morphology and early differentiation.

### 4.2. Bioink Preparation and Culture Conditions

Wharton’s jelly-derived stromal cells were used in all experiments. Cells were expanded in a growth culture medium consisting of DMEM:F12 (1:1) supplemented with 10% fetal bovine serum (FBS) and 10 ng/mL FGF2. Cells were passaged using a 10% TrypLE/1 mM EDTA solution in DPBS (all Gibco, Grand Island, NY, USA). Only cells between passages 4 and 8 (split ratios 1:2 or 1:3) were used throughout the study to ensure reproducibility and consistency of the experimental results. Stemness was routinely verified during cell expansion, and all experiments were performed using cells within this predefined passage range.

Source collagen (COL) type I, used to prepare the hydrogel matrix, was isolated from porcine skin using a 70% ethanol solution (*v*/*v*) (1 g skin/10 mL, 30 min), washed 3 times with water, and subsequently in an acetic acid solution at a ratio of 1:1000 (*v*/*v*) (1 g/20 mL, 48 h). COL in the collected supernatant was precipitated using a 0.1 M NaOH solution at a 6:1 ratio (*v*/*v*) up to neutral pH. The obtained pellets were then dissolved in a 1:1000 (*v*/*v*) acetic acid solution, frozen to −30°C, and lyophilized. All isolates were stored in a freezer at −25°C.

Lyophilized COL was sterilized by immersion in 70% ethanol solution (*v*/*v*) for 24 h. After removal from ethanol, the COL was freely dried in a laminar flow box. The sterilized COL was dissolved in 0.02 M acetic acid at a concentration of 30 mg/mL and stored at 4°C for 10 days. The COL suspension was homogenized using a disintegrator (10,000 rpm, 1 min) to obtain a solution.

To prepare the collagen bioink, the collagen solution was neutralized to physiological pH by mixing it with a 2× concentrated culture medium (2× concentrated DMEM:F12 with 20% FBS) and 1 M NaOH, with the required volume determined from titration curves [7]. A custom semi-automated mixing system was used to ensure precise volumetric dosing of all components [7]. This step is crucial to neutralize acidic collagen to a neutral pH of around 7. The neutralized collagen solution was then combined with a cell suspension prepared in 2× culture medium to obtain the final bioink. The detailed neutralization procedure and the resulting pH values after each neutralization step are described in our previous publication [7]. Final cell densities of 10 or 20 million cells/mL were used, depending on the experimental group.

To prevent premature gelation during preparation, all components, including collagen solution, culture media, syringes, and the final bioink, were maintained at approximately 5 °C using custom-designed cooling holders with circulating cold water.

Bioprinting was performed using a custom-built extrusion-based bioprinter equipped with a G20 nozzle (HotAIR, Ostrava, Czech Republic). Constructs with dimensions of 30 × 15 × 1.5 mm were printed onto sterile, tissue-treated glass coverslips (24 × 48 mm) at a feed rate of 2000 mm/min. The coverslips were secured onto a temperature-controlled print bed (heated to 37 °C) using a vacuum fixture.

Following bioprinting, samples were transferred to an incubator (37 °C, 5% CO_2_) to allow complete gelation. After approximately 5 min, constructs were immersed in culture medium and pre-incubated for 1 h.

Subsequently, samples were cultured under either static or dynamic conditions. Static samples were maintained in Petri dishes or well plates (TPP, Trasadingen, Switzerland). Dynamic cultivation was performed using custom-made polycarbonate bioreactors interconnected by silicone tubing and equipped with a dedicated insert to secure the constructs during perfusion.

**Figure 17:**
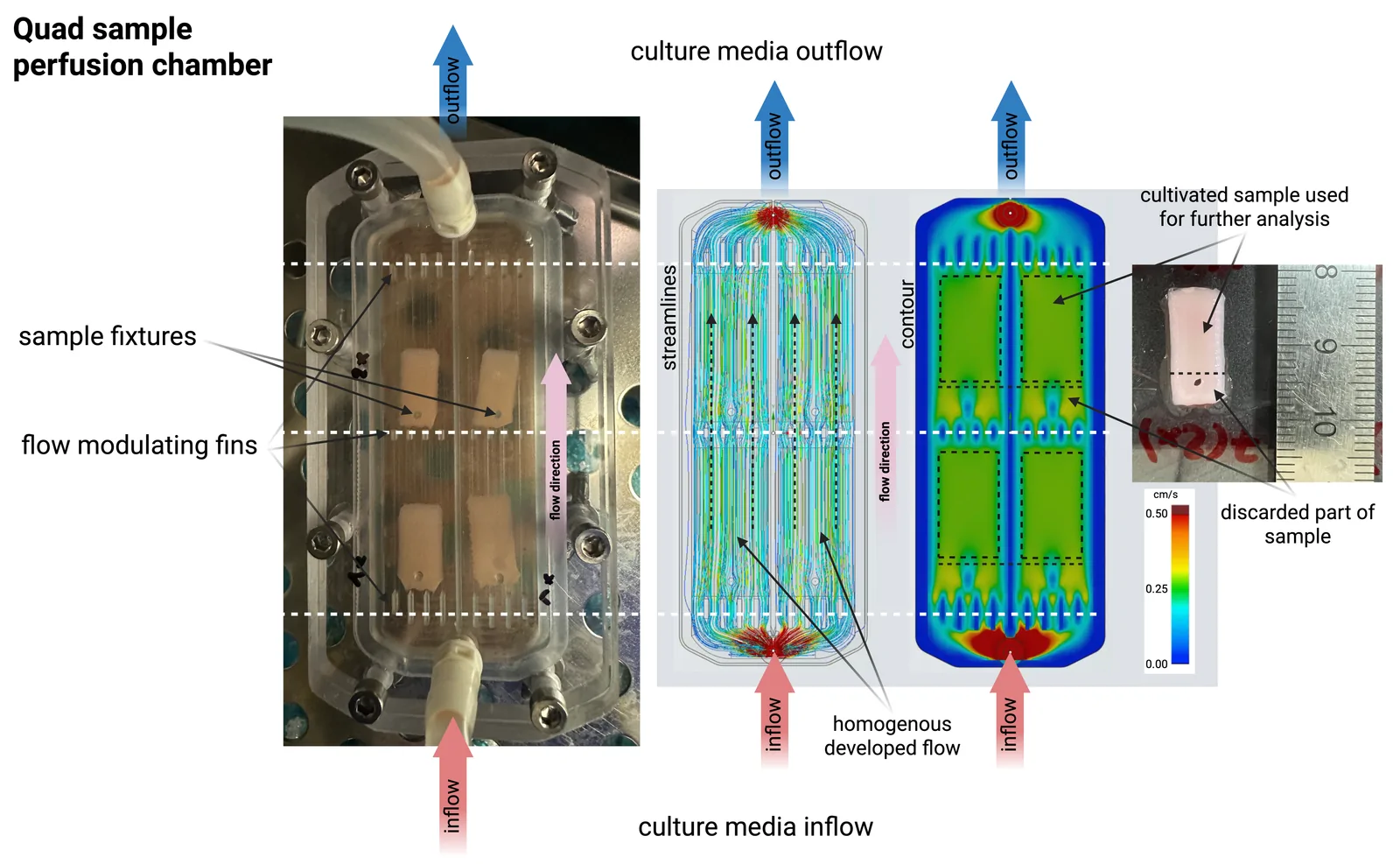
Design and flow characterization of the perfusion chamber used for dynamic cultivation. Created in BioRender. Matějka, R. (2026) https://BioRender.com/0qymett.

The bioreactors were connected to a custom perfusion system based on a syringe pump and pneumatically driven valves, generating continuous medium flow around the samples at a flow rate of 20 mL/min. The design of the perfusion chamber and the resulting flow distribution are illustrated in Figure 17. Computational fluid dynamics simulations confirmed a homogeneous flow profile across the sample region, supporting uniform exposure of the constructs to the culture medium during dynamic cultivation. Multiple chambers could be operated in series to enable parallel cultivation of multiple samples. The system operated as a closed perfusion circuit with automated medium exchange. All constructs were cultured for up to 8 days.

### 4.3. Immunofluorescence Assay

Filamentous actin (F-actin) was stained using ReadyProbes™ F-actin Phalloidin conjugates, and cell nuclei were counterstained with DAPI (ThermoFisher Scientific, Waltham, MA, USA). Samples were fixed with 4% (*w*/*v*) paraformaldehyde (PFA in PBS; ThermoFisher Scientific, Waltham, MA, USA) for 1–2 h and subsequently sectioned into thin slices. The sections were permeabilized with 0.1% Triton X-100 in PBS for 20 min and blocked with a blocking solution containing 1% bovine serum albumin (BSA) and 22.52 mg/mL glycine in PBST (PBS with Tween 20) for an additional 20 min. Immunostaining was performed either in a humidified chamber or directly in well plates at room temperature. Primary antibody incubation was carried out for 120 min, followed by a 3 min quenching treatment with SudanBlack (Ready ProbesTM Tissue Autofluorescence Quenching Reagent, Invitrogen, ThermoFisher, Scientific, Waltham, MA, USA) to reduce autofluorescence. Samples were then incubated with secondary antibodies for 30 min, thoroughly rinsed with PBS, and stored in PBS until imaging.

The stained cells were observed under a custom-built Thorlabs CERNA wide-field fluorescence microscope equipped with Olympus Plan Fluorite 10× objectives and a CCD camera from The Imaging Source and confocal imaging was performed using a Nikon CSU-W1 spinning disk system integrated into a Nikon Eclipse Ti2 inverted microscope (Nikon, Tokyo, Japan), equipped with a Yokogawa CSU-W1 module (Yokogawa, Tokyo, Japan) and dual PRIME BSI sCMOS cameras (Teledyne Photometrics, Tucson, AZ, USA). Samples were imaged using Nikon CFI Plan APO VC 10× and 20× dry objectives with a 25 µm pinhole disk in Z-stack acquisition mode (0–250 µm). Image acquisition and subsequent processing [35] were performed using Imaris 10.1.0 (Oxford Instruments, Abingdon, UK).

### 4.4. Scanning Electron Microscopy Images

To visualize the microstructure of collagen constructs, scanning electron microscopy (SEM) was performed using a Thermo Scientific Apreo S2 microscope (ThermoFisher Scientific, Waltham, MA, USA) operated in high-vacuum mode. Imaging was carried out using Everhart–Thornley in secondary electron mode at an accelerating voltage of 10 keV and magnifications ranging from 1000× to 20,000×.

Samples were fixed in a fixative solution (composition provided in Supplementary Materials part E) for 2 h at room temperature, followed by overnight fixation at 4 °C. After fixation, samples were rinsed in phosphate buffer supplemented with glucose and dehydrated through a graded ethanol–acetone series using a Leica EM TP tissue processor (Specion s.r.o., Prague, Czech Republic).

Dehydrated samples were dried using a Leica EM CPD300 critical point dryer, mounted on aluminum stubs with carbon adhesive tabs, and sputter-coated with a 13 nm platinum layer in an argon atmosphere using a Leica EM ACE600 system.

### 4.5. Remodeling Measurement Method

Sample remodeling was monitored throughout the cultivation period by image-based dimensional analysis. Daily photographs of each construct were acquired and analyzed using Fiji (ImageJ1.54f, National Institutes of Health, Bethesda, MD, USA) and custom-developed code to quantify temporal changes in construct geometry.

#### Domain Line Orientation Analysis of the Actin Cytoskeleton

Green-channel intensity was extracted from each image and lightly smoothed with a Gaussian kernel (σ = 0.5 px) to suppress single-pixel noise while preserving fine structural edges. Spatial gradients were computed using a 3 × 3 Sobel operator, yielding per-pixel gradient magnitude M and direction φ = arctan2(G_y, G_x). The local line orientation θ was derived as θ = (φ + 90°) mod 180°, since domain boundaries run perpendicular to the intensity gradient. Pixels with M below a threshold of 5 (out of 255) were excluded to suppress background regions with no structural content.

**Figure 18:**
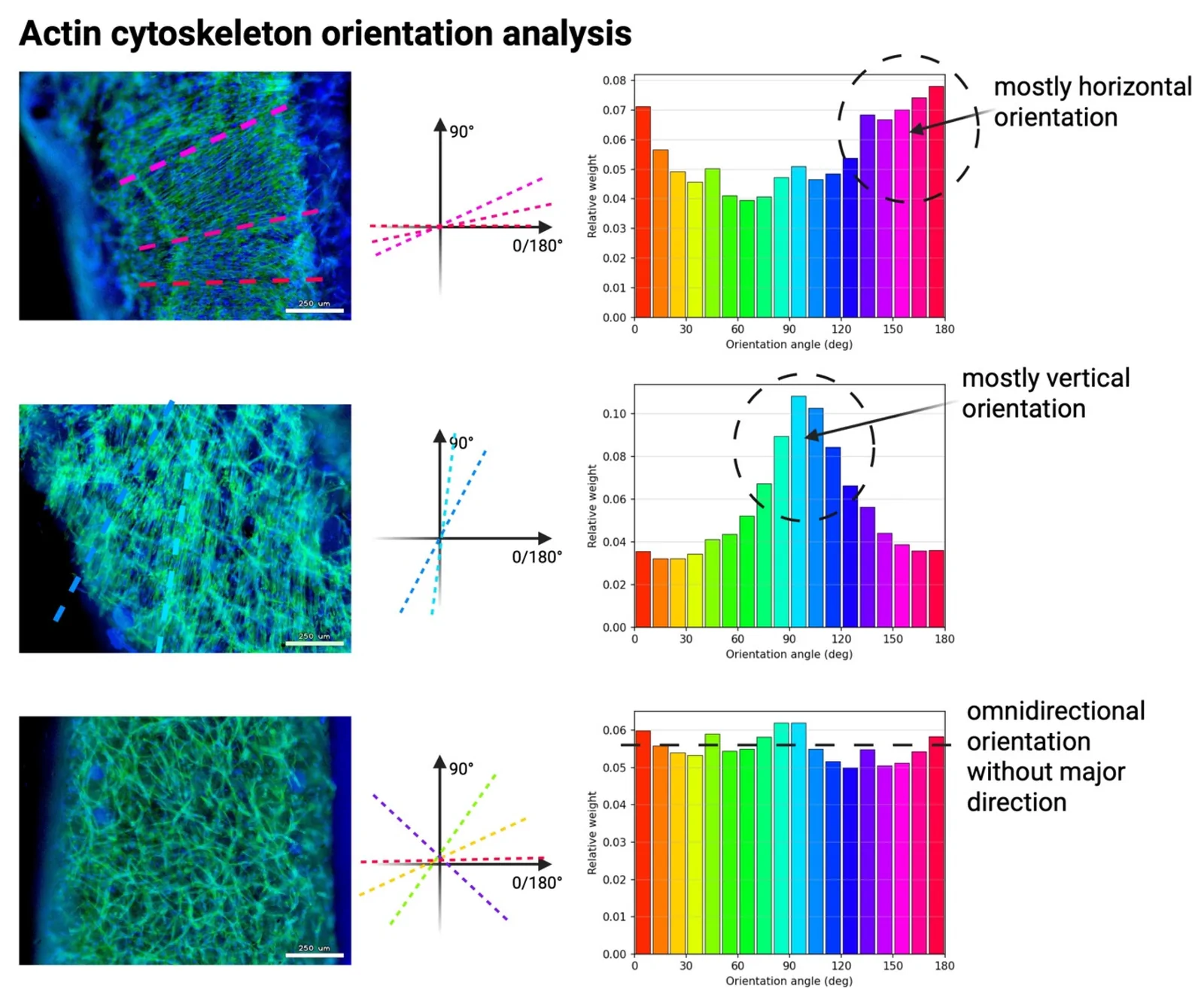
Orientation analysis of the actin cytoskeleton. Representative images and corresponding orientation histograms demonstrating horizontally aligned, vertically aligned, and isotropic cytoskeletal organization. Created in BioRender. Matějka, R. (2026) https://BioRender.com/2wv1rae.

A magnitude-weighted orientation histogram was constructed with 2.5° bins over the range [0°, 180°), where each pixel contributed a weight equal to its gradient magnitude M, so strongly defined boundaries dominated the distribution (Figure 18). The histogram was normalized to unit area to give relative orientation frequency. The three most prominent orientation angles were identified as the bins with the highest weighted frequency and are reported as the dominant domain line orientations.

### 4.6. Uniaxial Stretch Testing

To assess mechanical properties, a custom-built uniaxial testing machine was utilized. The moving platform featured a linear actuator equipped with a stepper motor and a trapezoidal lead screw with a 1 mm pitch, capable of generating peak forces up to 460 N (Nanotec Electronic GmbH & Co. KG, Feldkirchen, Germany). A prismatic linear coupling ensured precise movement. Samples were secured to thick, laser-cut paper sheets using cyanoacrylate adhesive. Strain measurements were obtained using an HT sensor TAS501N strain gauge, which has a 10 N range (HT Sensor Technology Co., Ltd., Xi’an City, China).

Strain analysis was performed by synchronizing a camera with the testing process and marking the samples with eyeliner for visual tracking. The distance between markers and sample width was measured to accurately calculate true stress and true strain. For each sample, three to four successful measurements were conducted following cultivation. Young’s modulus was determined from the linear portion of the stress–strain curve, and elongation was subsequently evaluated. Toughness was calculated as the area under the mean stress–strain curve up to failure by trapezoidal integration.

### 4.7. Statistical Evaluation

Linear regression with stepwise selection was used to assess the overall statistical significance of all variables together. The statistical significance level was set to 0.05 or better. Statistical evaluation was performed in R 4.6.1. More detailed results are available in Supplementary Materials parts A–E. Figures were created using Python3.13.13 with the Matplotlib 3.10.9 and Seaborn 0.13.2 modules.

## Figures and Tables

**Table 1:** Linear regression estimates for area remodeling.

	Estimate	Std. Error	*p*-Value
Cultivation days	−52.538	2.168	<0.001
Cell density	−50.980	7.497	<0.001
Static cultivation used	−62.593	8.571	<0.001
Proliferation medium used	10.23	7.73	0.187
(Intercept)	485.266	13.933	<0.001

Multiple R-squared: 0.71, Adjusted R-squared: 0.71.

**Table 2:** Estimates for Young’s modulus E (kPa).

	Estimate	Std. Error	*p*-Value
Cultivation days	−17.017	1.757	<0.001
Cell density	19.212	8.615	0.034
Static cultivation used	−3.737	8.998	0.068
Proliferation medium used	−40.007	9.091	<0.001
(Intercept)	151.320	17.063	<0.001

Multiple R-squared: 0.83, Adjusted R-squared: 0.81.

**Table 3:** Estimates for maximum strain (-).

	Estimate	Std. Error	*p*-Value
Cultivation days	0.114	0.011	<0.001
Cell density	0.134	0.053	0.018
Static cultivation used	−0.153	0.056	0.010
Proliferation medium used	−0.184	0.056	0.003
(Intercept)	0.134	0.053	0.687

Multiple R-squared: 0.81, Adjusted R-squared: 0.79.

**Table 4:** Estimates for maximum stress (kPa).

	Estimate	Std. Error	*p*-Value
Cultivation days	2.584	0.741	0.002
Cell density	2.921	3.632	0.042
Static cultivation used	−6.864	3.794	0.081
Proliferation medium used	−16.852	3.794	<0.001
(Intercept)	21.476	7.194	0.006

Multiple R-squared: 0.51, Adjusted R-squared: 0.44.

**Table 5:** Estimates for toughness (kJ/m^3^).

	Estimate	Std. Error	*p*-Value
Cultivation days	1.492	0.374	<0.001
Cell density	3.719	2.026	0.052
Static cultivation used	−6.631	1.917	0.002
Proliferation medium used	−8.575	1.937	<0.001
(Intercept)	−0.187	3.635	0.959

Multiple R-squared: 0.61, Adjusted R-squared: 0.56.

## Data Availability

Data are contained within the article.

## Supplementary Materials

The following supporting information can be downloaded at: https://www.mdpi.com/article/10.3390/gels12080698/s1.
