# Supplementary material for: Dynamic Perfusion and Cell Seeding Density Govern Remodeling and Mechanical Maturation of Bioprinted Collagen Constructs
Source: Gels. 2026 Aug 5;12(8):698. doi: 10.3390/gels12080698 (PMC13511877; doi:10.3390/gels12080698)
Supplement: Supplementary file 1 [file gels-12-00698-s001.zip › gels-4376539-supplementary.pdf]

Supplementary Figures part A – Cell growth curves

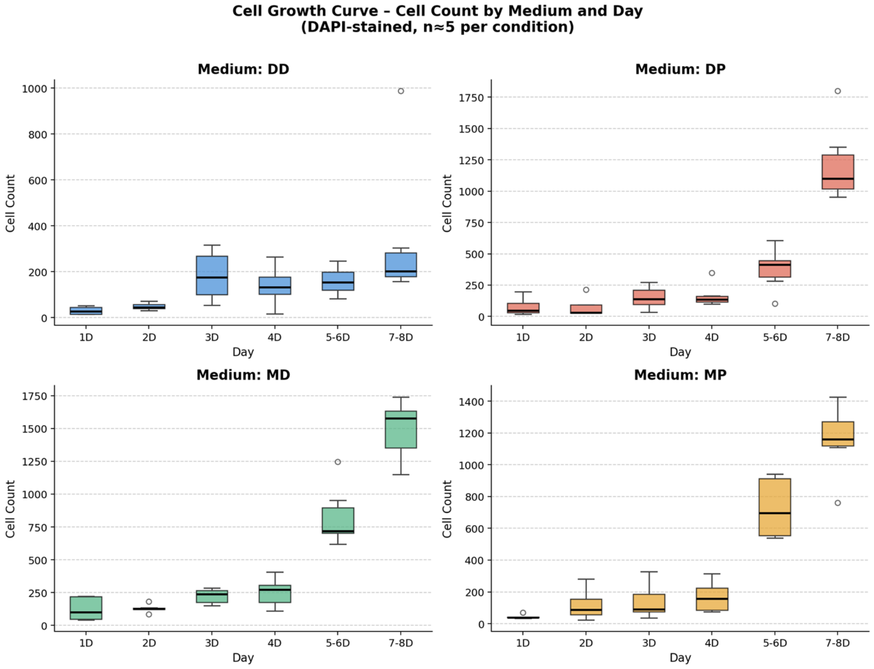

Figure S1. Growth curves of WJ-MSCs cultured in different media (DD, DP, MD, MP) over 7 days. Cell counts (DAPI staining, n = 5) show progressive increases in all conditions, with the highest proliferation observed in MD and MP media and consistently lower values in DD conditions.

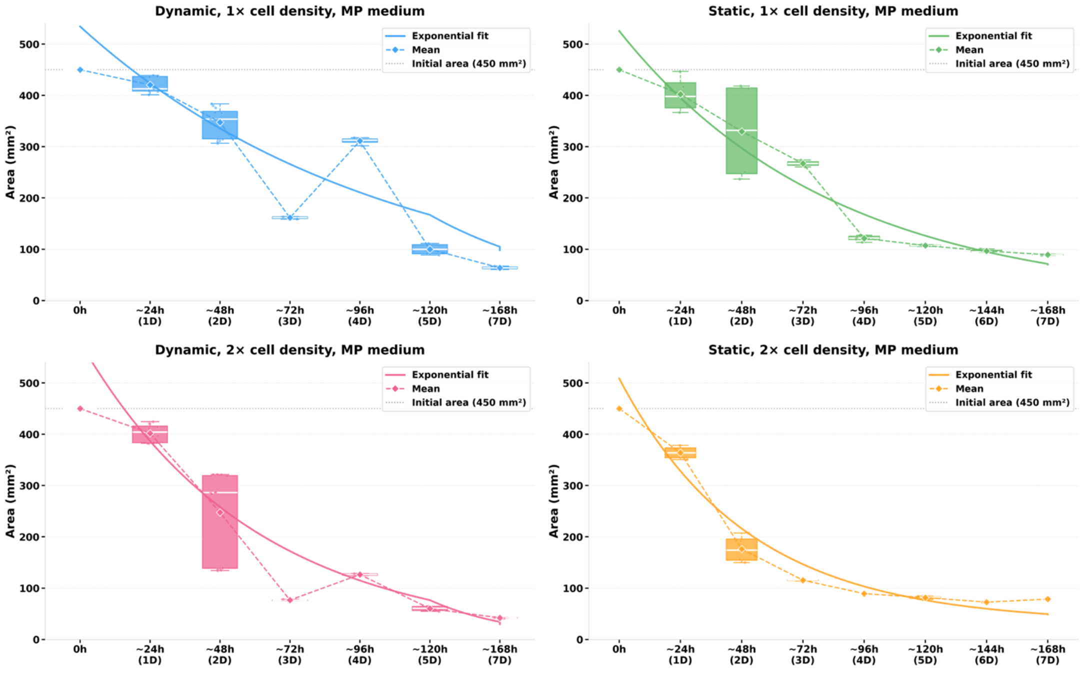

Figure S2. Dimensional remodeling of bioprinted collagen constructs cultured in MP medium under static and dynamic conditions at two cell seeding densities (1× (10 mill. cells/mL) and 2× (20 mill. cells/mL)). Mean values with exponential fits illustrate time-dependent contraction, with faster reduction in construct area observed at higher cell density and under dynamic cultivation.

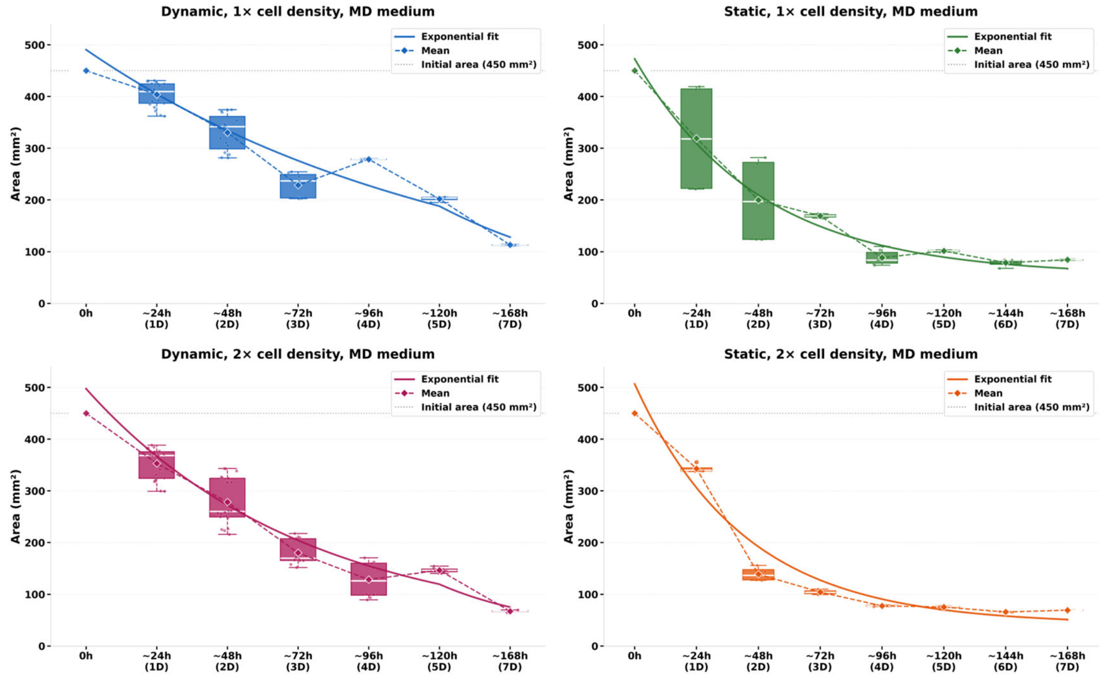

Figure S3. Dimensional remodeling of constructs cultured in MD medium under static and dynamic conditions at two cell seeding densities. Mean values with exponential fits show progressive contraction, with more controlled and gradual size reduction under dynamic culture compared to static conditions.

Supplementary Figures part B – all interval IF confocal images

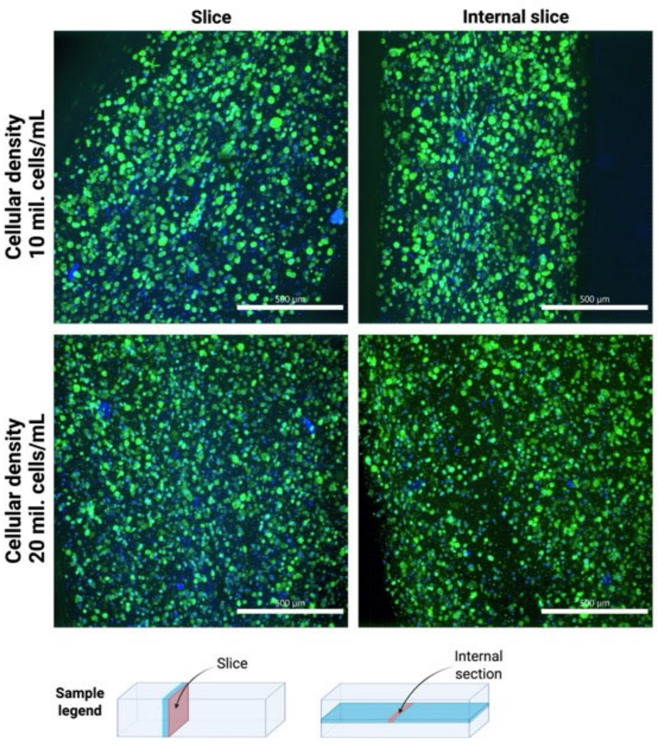

Figure S4. Fluorescence images of nuclei (blue) and actin cytoskeleton (green) showing initial cell distribution in collagen constructs immediately after bioprinting (day 0) at two cell seeding densities (10 and 20 mil. cells/mL). Images

are presented as surface slices and internal sections, as indicated schematically. Scale bars: 500 µm. Created in BioRender. Matějka, R. (2026) https://BioRender.com/arqa2lz

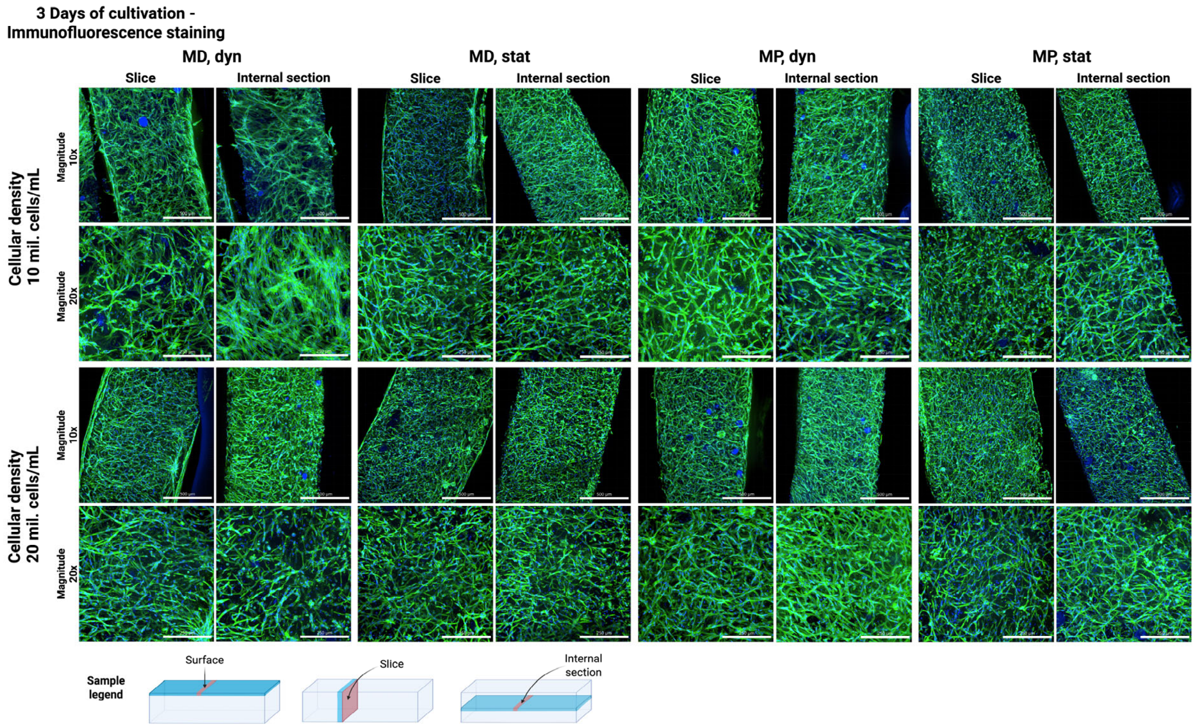

Figure S5. Immunofluorescence images of actin cytoskeleton (green) and nuclei (blue) in bioprinted collagen constructs after 3 days of cultivation under different culture conditions (MD, MP; static and dynamic) and cell densities (10 and 20 mil. cells/mL). Images show both surface slices and internal sections at two magnifications. Scale bars are indicated in each panel. Created in BioRender. Matějka, R. (2026) https://BioRender.com/8cjtri4

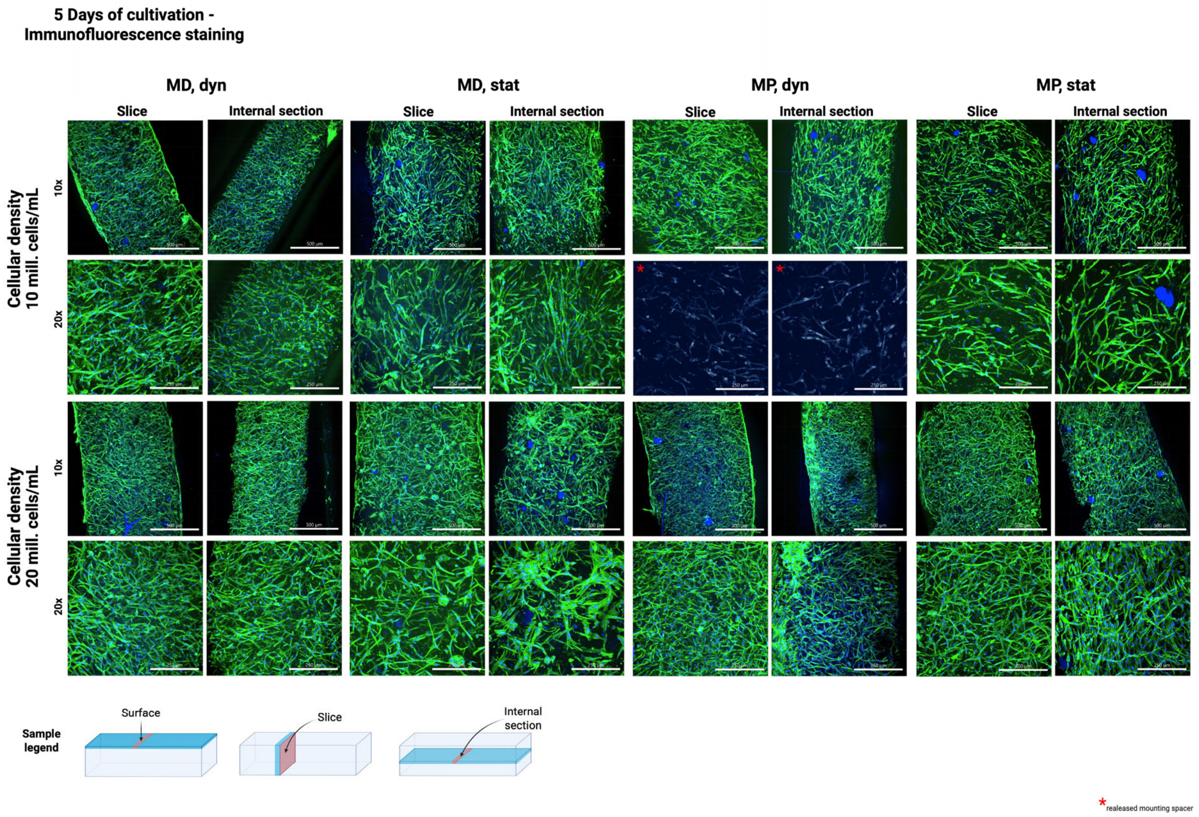

Figure S6. Immunofluorescence images of actin cytoskeleton (green) and nuclei (blue) in bioprinted collagen constructs after 5 days of cultivation under different culture conditions (MD, MP; static and dynamic) and cell densities (10 and 20 mil. cells/mL). Images show both surface slices and internal sections at two magnifications. Scale bars are indicated in each panel. Created in BioRender. Matějka, R. (2026) https://BioRender.com/xgsfuse

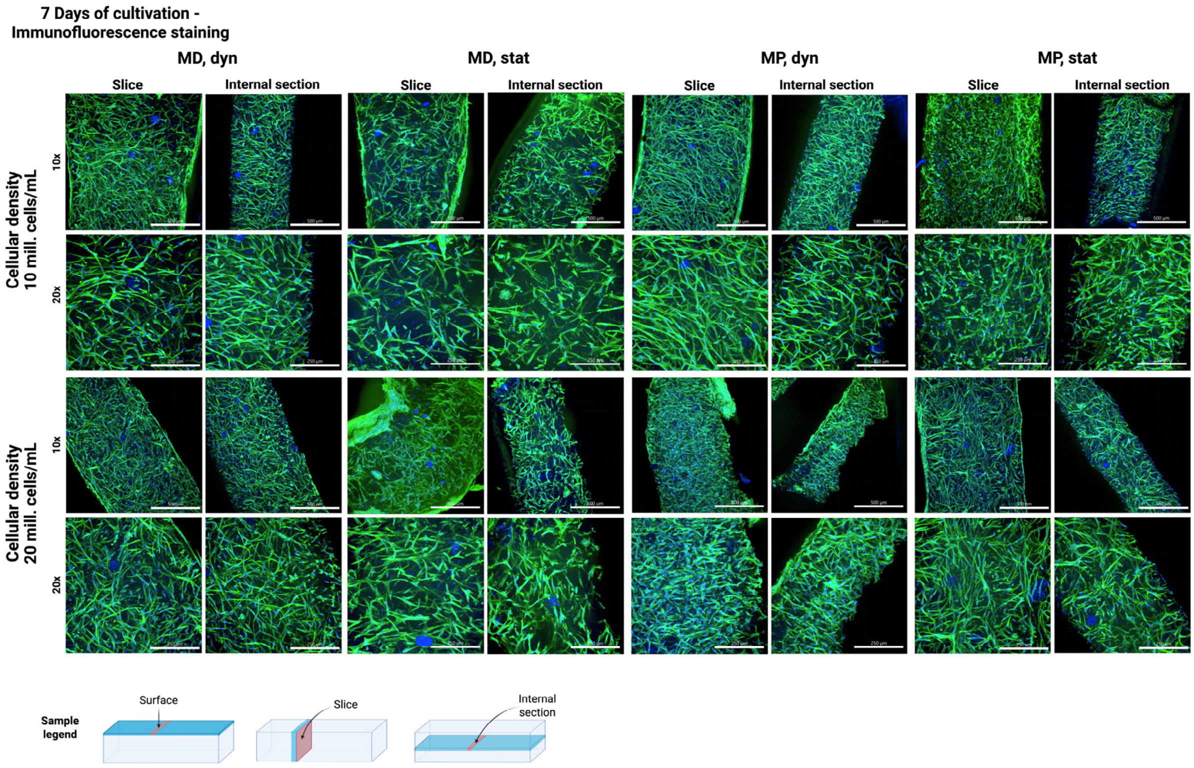

Figure S7. Immunofluorescence images of actin cytoskeleton (green) and nuclei (blue) in constructs after 7 days of cultivation under different culture conditions and cell densities. Scale bars are indicated in each panel. Created in BioRender. Matějka, R. (2026) https://BioRender.com/zn5a7s7

Supplementary Figures part C – all interval SEM images

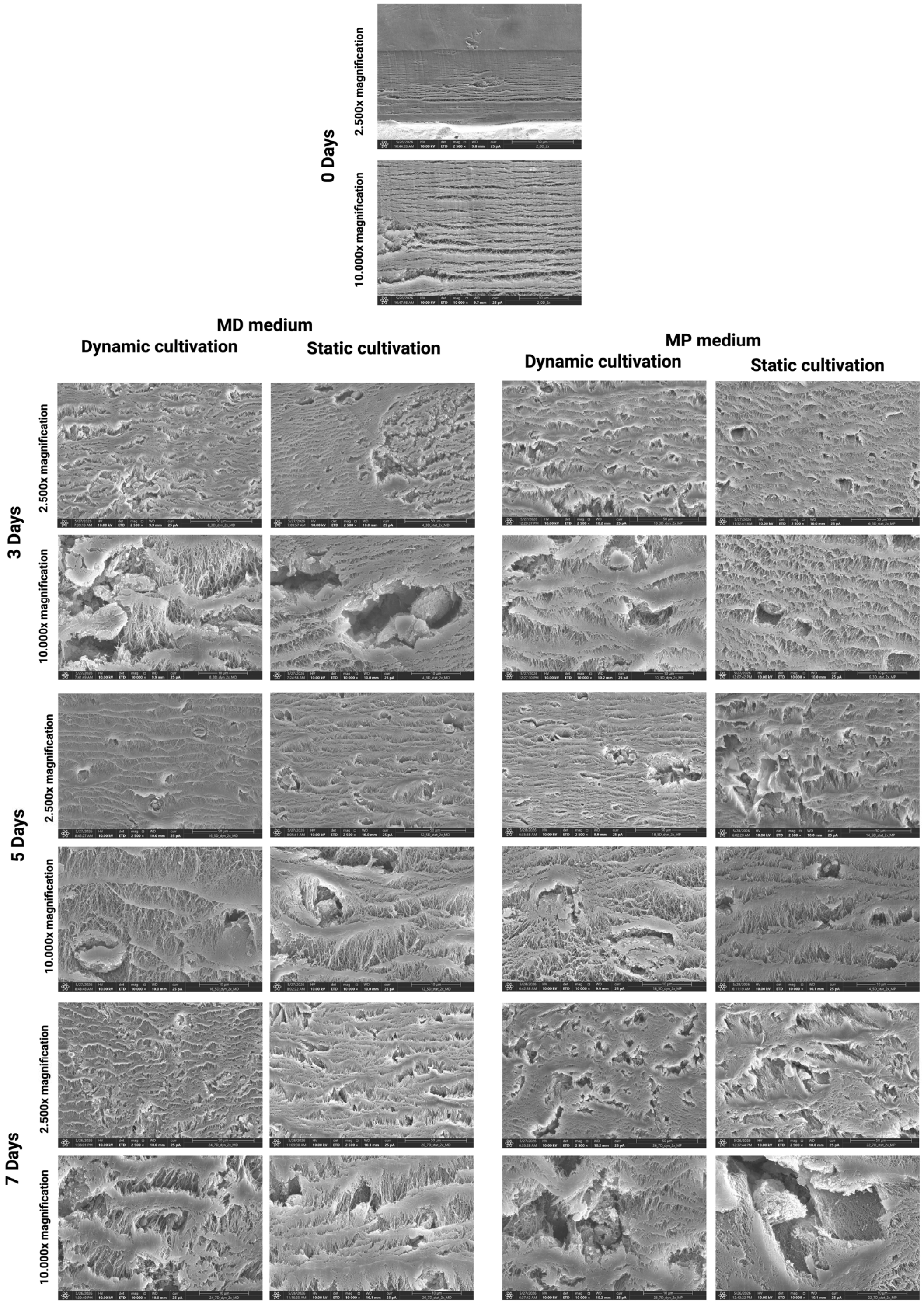

Figure S8. SEM images showing time-dependent evolution of collagen construct microstructure (0–7 days) under static and dynamic conditions in MD and MP media. Dynamic culture results in a more homogeneous organization, while static conditions lead to a heterogeneous structure and less controlled compaction. Created in BioRender. Matějka, R. (2026) https://BioRender.com/f6p95my

Supplementary Figures part D – raw and all modes stress-strain curves

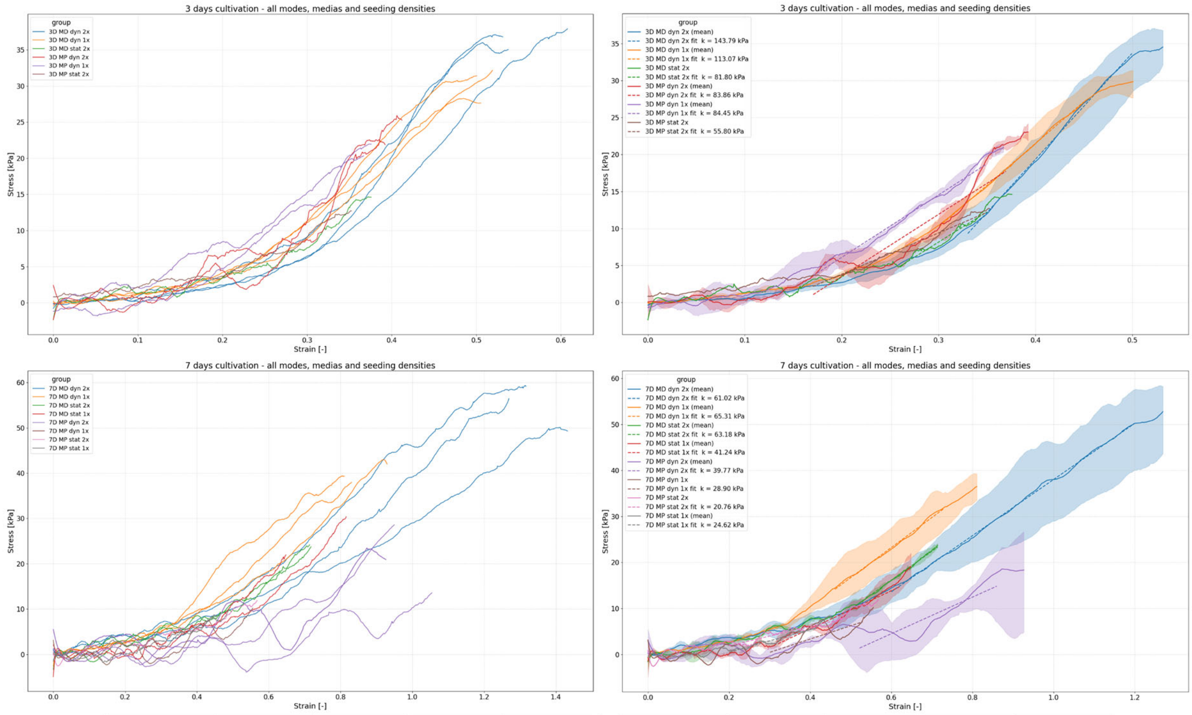

Group	E (kPa)	Max strain (-)	Max stress (kPa)	Toughness (kJ/m^3)
0D MD stat 1x	144,55	0,15	15,42	0,74
0D MD stat 2x	241,36	0,16	18,90	1,20
3D MD dyn 1x	113,05	0,51	30,62	5,44
3D MD dyn 2x	143,88	0,56	37,00	6,65
3D MD stat 2x	81,80	0,38	14,68	1,71
3D MP dyn 1x	84,40	0,37	21,15	2,67
3D MP dyn 2x	83,84	0,40	24,25	2,76
3D MP stat 2x	55,80	0,35	12,72	1,68

Group	E (kPa)	Max strain (-)	Max stress (kPa)	Toughness (kJ/m^3)
7D MD dyn 1x	65,30	0,86	40,10	12,92
7D MD dyn 2x	61,01	1,34	55,28	29,68
7D MD stat 1x	41,21	0,73	26,17	5,11
7D MD stat 2x	63,32	0,72	23,96	5,64
7D MP dyn 1x	28,90	0,56	10,20	1,04
7D MP dyn 2x	29,25	0,96	22,27	5,39
7D MP stat 1x	24,62	0,47	9,37	1,16
7D MP stat 2x	20,76	0,57	11,88	2,43

Figure S9. Overview of all measured mechanical data across all tested conditions, cultivation periods, and cell seeding densities. Individual stress–strain curves (left panels) and mean curves with min–max envelopes and linear fits (right panels) are shown for 3-day (top) and 7-day (bottom) constructs, encompassing all combinations of culture medium (MD and MP), cultivation method (dynamic and static), and cell seeding density (10 and 20 mil. cells/mL). The summary table provides the elastic modulus (E), maximum strain, maximum stress, and toughness for each group, including the day 0 measurement. Created in BioRender. Matějka, R. (2026) https://BioRender.com/2jj3zk9

Supplementary Figures part E – fixative for SEM

Preparation of Fixative Solution for SEM

Paraformaldehyde (2.5 g) was dissolved in 100 mL of distilled water under stirring at 60 °C. Once dissolved, 1 M NaOH was added dropwise until the solution became clear. After cooling to room temperature, 75 mL of 0.4 M phosphate buffer, 10 mL of 25% glutaraldehyde, and 0.75 mL of 0.5 wt.% $CaCl_2$ were added. The final volume was adjusted to 250 mL with distilled water, and the solution was thoroughly mixed and filtered. The pH was then measured and, if necessary, adjusted using 1 M NaOH to a final value of 7.2–7.5.
